# Multimode-enabled silicon photonic delay lines: break the delay-density limit

**DOI:** 10.1038/s41377-025-01820-2

**Published:** 2025-03-31

**Authors:** Shihan Hong, Long Zhang, Jiachen Wu, Yingying Peng, Linyan Lyu, Yinpeng Hu, Yiwei Xie, Daoxin Dai

**Affiliations:** 1 State Key Laboratory for Extreme Photonics and Instrumentation, College of Optical Science and Engineering, International Research Center for Advanced Photonics, Zhejiang University, Zijingang Campus, Hangzhou, 310058 China; 2 Intelligent Optics and Photonics Research Center, Jiaxing Research Institute, Zhejiang University, Jiaxing, 314000 China; 3 Ningbo Research Institute, Zhejiang University, Ningbo, 315100 China

**Keywords:** Silicon photonics, Slow light, Microwave photonics

## Abstract

Integrated optical delay lines have become imperative to meet the growing demand as large aperture antennas and high number of subarrays required for microwave beamforming, high-speed optical communication, and integrated quantum photonics. It is very challenging to achieve large delay ranges, small footprints, and broad bandwidths simultaneously due to the strong trade-off between the propagation loss and the group refractive index of optical waveguides. In this paper, we propose and experimentally demonstrate multimode-enabled silicon photonic delay line for the first time, which breaks the delay-density limit of singlemode waveguide spirals, towards a broadband, mm^2^-scale, and ultra-large time delay. By demonstrating low-loss-propagation possibilities for different polarizations and modes, we introduce a novel multimode delay unit by integrating the mode (de)multiplexers and the ultralow-loss multimode waveguide spiral supporting the TE_0_, TE_1_, and TE_2_ modes propagating in parallel. The measured propagation losses for the TE_0_, TE_1_, and TE_2_ modes are 0.2 dB/cm, 0.31 dB/cm, and 0.49 dB/cm, respectively. In this way, the *highest* line delay-density of 376.9 ps/cm and low delay loss of 0.004 dB/ps are achieved. Furthermore, we implement a 7-bit tunable multimode photonic delay line and experimentally demonstrate an ultra-large delay range of 12.7 ns with a delay resolution of 100 ps and within an ultra-compact footprint of 3.85 mm^2^, enabling a delay density over 3299 ps/mm^2^, showing the *largest* delay range and the *highest* delay density among on-chip delay lines reported to date, to the best of our knowledge.

## Introduction

Optical delay lines, which buffer optical signals by slowing the speed of light or extending lengths of waveguide, are one of the most essential tools in but not limited to optical communication, microwave photonics, coherence detection, quantum computing, and optical measurement^1–5^. Conventional fiber-based optical delay lines^6–8^, typically constructed from low-loss fibers and benchtop switches to achieve long-range delay, suffer from bulky components and the need for precise cleaving for accurate delays. These characteristics make them inadequate for the growing demands of compact, mobile and specific applications, such as optical frequency domain reflectometry (OFDR) for remote sensing^9^, biological optical coherence tomography (OCT) for medical diagnosis^10^, microwave beamforming^11,12^ for reducing beam-squint and quantum communications for phase decoding^13^. In contrast, on-chip optical delay lines^4^ offer great advantages including compact footprints, high delay accuracy/stability as well as low power consumption and cost, and thus have garnered attention from both academia and industry. So far, several representative on-chip optical delay lines based on different platforms have been proposed and experimentally demonstrated in silicon on insulator (SOI)^14–17^, silicon nitride (SiN)^18,19^, silica (SiO_2_)^20^ and thin film lithium niobate (TFLN)^21^. Generally speaking, the optical waveguides with a lower index-contrast Δ can offer a lower propagation loss^22^, while they usually have to be designed with a larger bend radius for minimizing the bending losses as well as a larger separation between adjacent waveguides for avoiding the evanescent coupling. In addition, they often have a lower group refractive index *n*_g_, which necessitates longer waveguides to achieve the desired time delay. Therefore, the optical delay lines often have large footprints when using low-Δ waveguides. In order to achieve long time delay in compact footprints, high-Δ optical waveguides are usually preferred and ultra-high-Δ silicon photonic waveguides stand out as a promising solution with unique advantages^23–26^. Besides, the CMOS compatibility of silicon photonics enables the integration of optical delay lines with the arrays of modulators and photodetectors monolithically, facilitating cost-effective and high-volume manufacturing of systems-on-chip with optical delay lines. These advantages position silicon photonics as an ideal platform for building high-performance delay line systems.

To date, however, none of the on-chip optical delay lines demonstration can break the technical bottleneck to achieve broadband, ultra-compact footprint down to mm^2^-scale and large time delay of >10 ns simultaneously. These are, however, crucial requirements towards an forceful tool to realize large aperture antennas and arrays for microwave beamforming with a high number of subarrays requiring delay lengths in the tens of nanosecond scale^18,27–31^, OFDR^32^ and OCT^5,33^ for extended detection range and great imaging depth, optical communication for high speed^34,35^, and integrated photonic quantum technologies for receivers and optical equivalent random access memory^36^. As is known to all, the delay time τ in an optical waveguide is given as $\tau =-\frac{\partial \varphi (\omega )}{\partial \omega }=\frac{{n}_{g}L}{c}$, where *L* is the waveguide length and *c* is the speed of light in the vacuum^4^. Therefore, the delay enhancement can be achieved by increasing group refractive index *n*_g_ or extending the propagation length *L*.

In principle, one can achieve a significant increase in *n*_g_ by introducing the slow light effect, and currently there have been some on-chip resonant delay line structures reported by using e.g., microring resonators^15,37–39^ (MRRs), cascaded MRRs^40–43^, Bragg gratings^44–46^ and photonic-crystal waveguides^47–50^, which generally achieve enhanced delays at the resonant wavelengths. Besides, due to the slow light effect, slight changes in the effective refractive index *n*_eff_ of the optical waveguide via thermo-/electro-optic effects can cause significant variations in *n*_g_, thereby enabling continuous delay varying. However, their delay-bandwidth product is constant and thus their working bandwidth is usually very limited. Take all-pass MRR as an example, which has a bandwidth of only 0.64 GHz for a 1 ns time delay, since the delay bandwidth product is 2∕π^4^.

Another promising approach is to achieve digitally tunable time delay^14–21,27–30^ by routing the light propagation to go through the waveguide delay lines with different physical lengths with the assistance of on-chip optical switches (OS) in cascade. Since the group refractive index *n*_g_ of a regular optical waveguide changes minimally within a certain wavelength range, the delay is essentially wavelength-insensitive. In order to characterize the time delay capability and the footprint compactness for an optical delay line, here we introduce a figure of merit called as the delay density *D*_d_, which is defined as the time delay per unit area (ps/mm²). Generally speaking, the achievable delay density is mainly determined by the line delay-density *D*_d_L_ and the delay loss *L*_d_. Here the line delay-density *D*_d_L_ is defined as the ratio of the time delay to the waveguide length with the unit of ps/cm, while the delay loss *L*_d_ is defined as the ratio of the propagation loss to the delay time with the unit of dB/ps. We compare the delay density *D*_d_ of single waveguide spirals for the state-of-the-art structures/platforms in Supplementary information, Section S3.1. It shows that a single singlemode SOI photonic waveguide spiral can achieve the present delay density limit up to 4400 ps/mm^2^, but the unacceptable propagation loss of 2 dB/cm prevents it to achieve a time delay more than 1 ns. In contrast, a broadened SOI photonic waveguide spiral^17^ and a SiN photonic waveguide spiral^19^ exhibit significantly lowered propagation losses of 0.1–0.3 dB/cm, allowing to achieve an on-chip time delay as large as 10 ns, while their delay densities are less than 3000 ps/mm² because their bending radii are relatively large and group refractive indices are relatively low. Therefore, it is still very challenging to simultaneously achieve high delay density *D*_d_ as well as low delay loss *L*_d_, which is one of the primary tasks for developing large-scale on-chip photonic systems with the array of optical delay lines.

In this paper, a multimode-enabled low-loss silicon photonic delay line to break the delay density limit is proposed by introducing ultralow-loss multimode photonic waveguides and multiplexing more than one mode-channel as well. In particular, we build a novel multimode delay unit (MDU) by integrating a pair of mode (DE)multiplexers (MUXes) with the ultralow-loss multimode waveguide spiral (MWS) to facilitate the TE_0_, TE_1_ and TE_2_ modes propagating in parallel, which greatly enhances the high delay density *D*_d_ and achieves a low delay loss *L*_d_ as well. In the present case, the MDUs and a 7-bit tunable multimode photonic delay line were fabricated with standard 220-nm-thick SOI multi-project-wafer (MPW) processes. The measured propagation losses of the TE_0_, TE_1_, and TE_2_ modes are 0.20 dB/cm, 0.31 dB/cm, and 0.49 dB/cm, respectively. Accordingly, we achieve the *highest* line delay-density of 376.9 ps/cm and low delay loss of 0.004 dB/ps experimentally as predicted by our theoretical analysis. The fabricated 7-bit tunable multimode photonic delay line has an ultra-compact footprint of 3.85 mm^2^ and exhibits an ultra-large delay range of 12.7 ns with a delay resolution of 100 ps. To the best of our knowledge, the present tunable photonic delay line shows the *largest* delay range and *highest* delay density (3299 ps/mm^2^) among the on-chip photonic delay lines reported to date.

## Results

### Principle and design

**Fig. 1:**
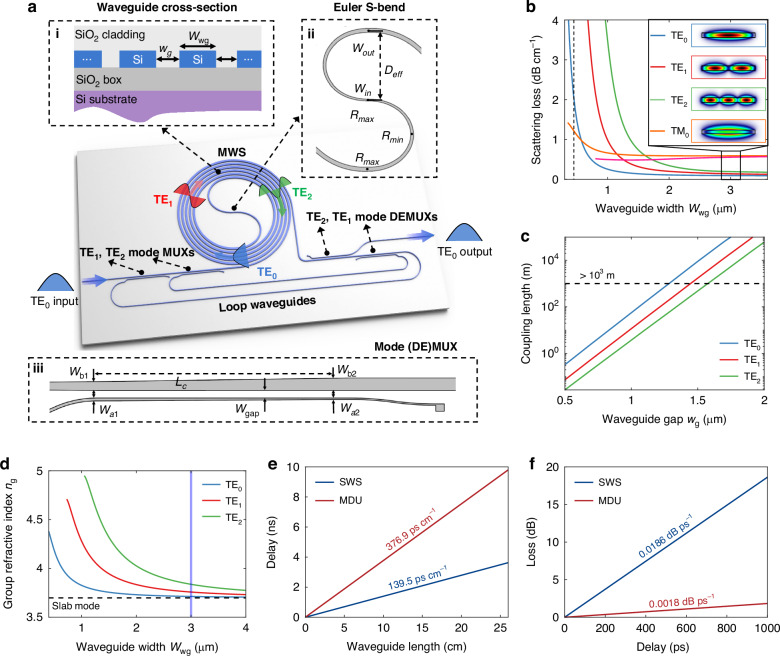
Multimode-enabled silicon photonic MDU. **a** Schematic of the proposed MDU, which consists of singlemode input/output waveguides, multimode waveguide spiral (MWS), Euler S-bend, TE_1_, TE_2_ mode MUXes/DEMUXes and two loop waveguides. **b** Calculated scattering losses for the TE_0_, TE_1_, TE_2_, and TM_0_ modes as the waveguide width *W*_wg_ varies. **c** Calculated coupling lengths for the TE_0_, TE_1_, and TE_2_ modes as the waveguide gap *w*_g_ varies. **d** Calculated group refractive indices *n*_g_ for the TE_0_, TE_1_, and TE_2_ modes as the waveguide width *W*_wg_ varies. **e** Simulated line delay-density and **f** simulated delay loss for the singlemode waveguide spiral (SWS) and MDU with the TE_0_, TE_1_, and TE_2_ modes

Figure 1a illustrates the schematic layout of the proposed multimode delay unit (MDU), which consists of singlemode input/output waveguides, multimode waveguide spiral (MWS) based on Archimedean spiral structure, Euler S-bend, mode MUXes, DEMUXes and two loop waveguides. As shown in Fig. 1a, the spiral waveguide is broadened to be beyond the singlemode regime for supporting multiple guided-modes and low-loss propagation. Additionally, the waveguide gap *w*_g_ between adjacent waveguides in the spiral is chosen optimally to avoid coupling crosstalk and to achieve compact footprint simultaneously. At the center of the MWS, we introduced a Euler S-bend^17^ to achieve an ultra-compact size and an ultralow bending loss as well as negligible higher-order-mode excitation for the three involved mode-channels. Detailed design and simulation results of the Euler S-bend [whose key parameters are given in the inset II of Fig. 1a] are given in the Supplementary information, Section S4.1. The present three-channel mode (DE)MUXes, as shown in inset III of Fig. 1a, are designed with adiabatic direction couplers^51–53^, and more details are given in Supplementary information, Section S4.2. Finally, adiabatic tapers are implemented between each mode (DE)MUX and the waveguide spiral to ensure lossless connections between the waveguides with different widths. The MDU realizes the TE_0_, TE_1_, and TE_2_ modes propagating in parallel by converting orthogonal mode at corresponding mode (DE)MUX and circulating propagation using loop waveguide. The details about the light propagation of three guided-modes in the MDU are given in Supplementary information, Section S5. In this way, light triply goes through the MWS in the way carried by different guided-modes, accordingly enhancing the effective group index *n*_g_ of this waveguide spiral to the sum of *n*_g_TE0_, *n*_g_TE1_, and *n*_g_TE2_, and significantly increasing the total delay time. Note that the present multimode photonic waveguide delay line can be further extended with more higher-order modes to increase the line delay-density by introducing higher-order mode MUXes and DEMUXes. The discussion on the extensibility of higher-order modes is detailed in Supplementary information, Section S3.3.

As it is well known, reducing the waveguide propagation loss is critical for determining the upper limit of the time delay of on-chip delay lines. This is particularly challenging for multimode photonic waveguide delay lines, as it requires simultaneously to achieve low losses for all the multiple modes involved in propagation. For the current MPW standard process for silicon photonics, material-related losses are already minimized to be negligible (<0.01 dB/cm)^54^, and the unavoidable scattering loss due to the waveguide surface roughness becomes the primary loss source (at the level of 2–4 dB/cm). In MPW standard processes, the user can do nothing to reduce the roughness of the waveguide surfaces. Alternatively, one can minimize the scattering loss by optimizing the mode field to lower the overlap between the mode field and the rough waveguide surfaces. As demonstrated by our previous work^17,54^, broadened waveguides are employed to ensure on-chip low-loss waveguide propagation. Moreover, the broadened waveguides can potentially work very well with the fundamental modes as well as the higher-order modes if the inter-mode crosstalk can be sufficiently low, which is feasible with careful design for the multimode spiral structure. In this work, we introduce Archimedean spiral and Euler S- and 90°- bends to implement smooth transitions in curvature or width at the regions where there is some risk to see inter-mode coupling.

One should note that the selection of polarizations and modes is the fundamental for ensuring low-loss propagation in MDU. Here, a three-dimensional volume current method is used to quantify the scattering losses at the sidewalls and top/bottom surfaces of the waveguide for different guided-modes^17,54–56^ (see the details in Supplementary information, Section S6.1). The calculated scattering losses for the TE_0_, TE_1_, TE_2_, and TM_0_ modes at varying waveguide width *W*_wg_ are shown in Fig. 1b using the parameters in^56^. Here the TE_0_ and TM_0_ modes are taken as examples to analyze the propagation losses of two polarization modes. Although the TM_0_ mode has a lower propagation loss of ~1.2 dB/cm than the TE_0_ mode (~2 dB/cm) at the singlemode regime, the TM_0_ mode has much higher scattering losses than the TE_0_ mode for a broadened waveguide due to much stronger filed intensity at the top/bottom surface. The detailed analysis of the scattering losses for different polarizations and modes are given in Supplementary information, Section S6.2. Therefore, the fundamental and higher-order modes of TE-polarization are considered in this paper. Particularly, here we employ the TE_0_, TE_1_, and TE_2_ modes for the MDU, while their mode profiles and scattering losses are shown in Fig. 1b. It can be seen that these three guided-modes have low losses of *L*_TE0_ = 0.14 dB/cm, *L*_TE1_ = 0.21 dB/cm, *L*_TE3_ = 0.34 dB/cm when the waveguide width is chosen as wide as 3 μm. In addition, to avoid the coupling crosstalk between adjacent waveguides in the spiral, especially for the higher-order modes (which have weaker mode confinement), the waveguide gap should be chosen to be large sufficiently, as shown in Fig. 1c. Here, when the waveguide gap is chosen as *w*_g_ = 1.8 μm, the coupling lengths for all these three modes are more than 1000 m, indicating the evanescent coupling is low enough to avoid the crosstalk between the adjacent waveguides. One should note that higher-order modes have higher field intensity at the waveguide surface, leading to slightly higher scattering losses, as shown in Fig. 1b. In this paper, we consider the design with the TE_0_, TE_1_, and TE_2_ modes while it is possible to be scaled with other higher-order mode-channels.

Figure 1d shows the calculated group refractive indices *n*_g_ of these three guided-modes. As the core width increases, *n*_g_ gradually approximates that of the slab modes in a planar waveguide with a core height *H*_co_ = 220 nm. When *W*_wg_ = 3μm, one has *n*_g_ = 3.712, 3.767 and 3.836 for the TE_0_, TE_1_, and TE_2_ modes, respectively. The discussion about the bandwidth of the delay is detailed in Supplementary information, Section S3.2. Figure 1e shows the calculated line delay-density *D*_d_L_ for a singlemode waveguide spiral (SWS, *W*_wg_ = 0.45 μm) and the present MDU according to the calculated group refractive index *n*_g_. It can be seen that the present MDU exhibits a line delay-density as high as 376.9 ps/cm, which is 2.7× higher than that of the 0.45-μm-wide singlemode waveguide and 3.05× higher than that of the 3.0-μm-wide broadened waveguide working with the TE_0_ mode only. Moreover, the delay losses *L*_d_ for the SWS and the MDU are illustrated in Fig. 1f. It can be seen that the present MDU achieves an ultralow delay loss of 0.0018 dB/ps, which is an order of magnitude smaller than that for an SWS. Thanks to the high line delay-density and ultralow delay loss, it is possible to develop on-chip tunable photonic delay lines beyond 10 ns within an extremely compact footprint, laying a solid foundation for on-chip large-scale and multi-array systems with optical delay lines.

### Experimental characterization

**Fig. 2:**
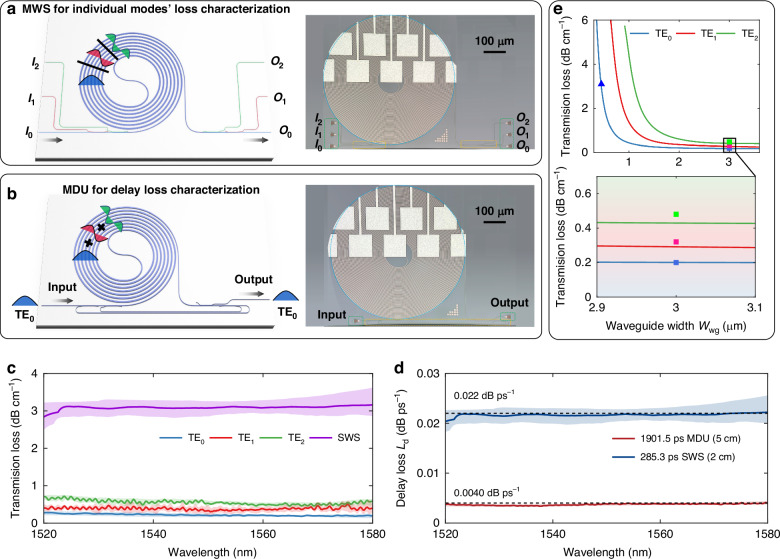
Characterization of the propagation losses for three individual modes and delay losses on 10 chips. **a**, **b** Schematic configurations and microscope images of the fabricated (**a**) MWS for characterizing the propagation losses of TE_0_, TE_1_, and TE_2_ modes and (**b**) MDU for characterizing delay losses. **c** Measured transmission losses of the TE_0_, TE_1_, TE_2_ in MWSs and TE_0_ in SWSs on 10 chips. **d** Measured delay losses of MDUs and SWSs on 10 chips. **e** Fitting of theoretical scattering losses and measured results

The proposed MWS/MDU and tunable multimode silicon photonic delay line were fabricated at the MPW foundry with the standard processes. Figure 2a shows the structures to individually characterize the propagation losses of each mode in the developed MWSs. This structure in Fig. 2a involves an MWS integrated with mode MUXes/DEMUXes, allowing to selectively excite the TE_*i*_ mode in the MWS by launching light from input port I_*i*_ and monitoring the output power from output port O_*i*_ one by one, where *i* = 0, 1, 2. The measured results for each mode are normalized with respect to the referenced mode (DE)MUXes on the same chip. Detailed measurement results for the mode (DE)MUXes for the TE_0_, TE_1_, and TE_2_ modes can be found in Supplementary information, Section S4.2. Figure 2b shows the structure of the proposed MDU, which consists of a pair of mode (DE)MUXes connected with loop waveguides to support the mode-division-multiplexed light propagation in the MWS simultaneously. Note that the losses of the mode (DE)MUXes and the loop waveguides are included when characterizing the total delay loss for the present MDU. The microscope images of the fabricated MWSs/MDUs with a 5-cm-long waveguide spiral are also shown in Fig. 2a, b. Figure 2c gives the measured transmission losses of the TE_0_, TE_1_, TE_2_ modes in the MWSs and the TE_0_ mode in the SWSs on 10 chips, showing that the measured propagation losses at 1550 nm are 0.2, 0.31, and 0.49 dB/cm for the TE_0_, TE_1_, and TE_2_ modes, respectively, which are 1/15, 1/10, and 1/6 of that (~3.1 dB/cm) for the SWSs fabricated on the same chip. The MDUs’ measured results shown in Fig. 2d reveal an average delay loss of 0.0040 dB/ps, which is 2/11 of the delay loss (~ 0.022 dB/ps) for SWSs on the same chip. One should notice that the total loss of the MDU is slightly higher than the sum of the measured individual losses for the TE_0_, TE_1_ and TE_2_ modes, because the losses from the mode (DE)MUXes and the loop waveguides are included. By excluding the loss of the mode (DE)MUXes, the delay loss for the MWS alone is as low as 0.0026 dB/ps, which is ~1/9 of that for the SWS. This is similar to the simulation delay losses of 0.0018 dB/ps and 0.0186 dB/ps respectively for the MDU and the SWS. The data transmissions of the MWS and the MDU are shown in Supplementary Information, Sections S7, indicating negligible inter-mode crosstalk within the delay line. With these measured data about the propagation losses for all these three guided-modes, it is possible to extract the waveguide’s surface/sidewall roughness (*σ*_sidewall,_ *σ*_surface_) and the correlation length *L*_c_sidewall_ and *L*_c_surface_. Figure 2e shows the fitting result obtained from the three-dimensional volume current method^17^, and one has the estimated roughness values of *σ*_sidewall_ = 3.4 nm, *σ*_surface_ = 0.42 nm, *L*_c_sidewall_ = 60 nm and *L*_c_surface_ = 60 nm. Noticed that both the MWS and SWS fabricated in this MPW batch exhibited higher propagation losses than those with the same structures in previous MPW runs, which might be due to some unexpected fabrication issue happening in this batch. We believe that further reduction of the delay loss is expected in the future MPW fabrication.

**Fig. 3:**
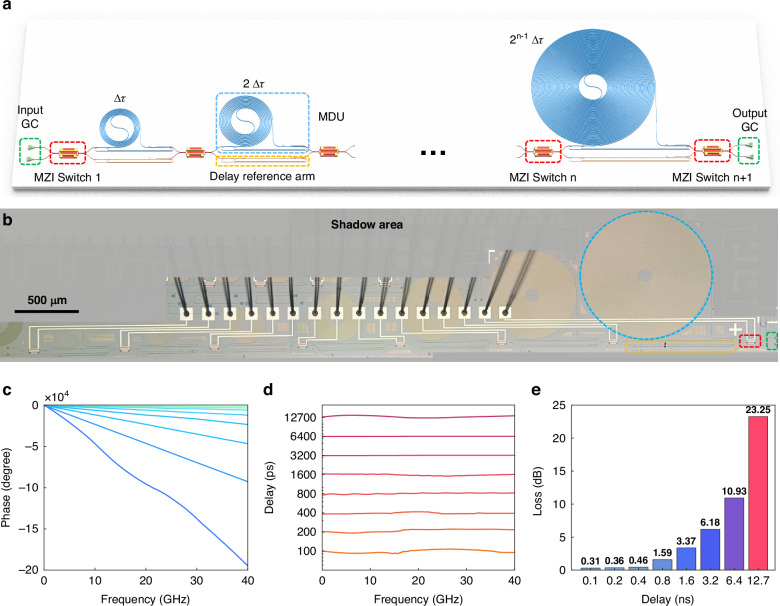
Experimental results of the tunable multimode photonic delay line. **a** Schematic configuration and **b** microscope image of the fabricated 7-bit tunable multimode photonic delay line. The measured microwave (**c**) phase response and **d** delay response. **e** Measured excess losses for different delay states

Ultimately, a 7-bit tunable multimode photonic delay line is developed with our proposed MDUs and optimized 2 × 2 optical MZI switches, as depicted in the schematic configuration and microscope image shown in Fig. 3a, b. The delay reference arm comprises a pair of mode (DE)MUXes and straight waveguides. Here the optical MZI switches are based on MZIs^57,58^, equipped with silicon slabs and broadened phase shifters to achieve high speed and calibration-free opeation. More details of the optical MZI switch can be found in Supplementary information, Section S4.3. The longest delay spiral to providing a delay of 6400 ps is 17-cm-long and occupies a footprint of 0.87 mm^2^. The delay time were measured via microwave responses, with the experimental setup described with details in Supplementary Information, Section S8. By switching to different dealy paths, the measured microwave phase response and the corresponding delay response are shown in Fig. 3c, d. Utilizing the high line delay-density and low delay loss of the MWS, our tunable multimode photonic delay line achieves, to the best of our knowledge, the highest on-chip delay of 12.7 ns, with a resolution of 100 ps and a compact footprint of 3.85 mm^2^. The delay profile at 12.7 ns exhibits slight fluctuations, primarily due to insufficient output microwave power. The measured optical loss for the cases with different delays are shown in Fig. 3e, showing a maximum loss of 23.2 dB for the longest path (i.e., 33.67 cm). The measured overall transmission for the tunable multimode photonic delay line is detailed in Supplementary Information, Section S9.

## Discussion

In this work, we have proposed and experimentally demonstrated a multimode-enabled silicon photonic delay line that break the delay density limit of on-chip delay lines, towards a broadband, tunable, mm^2^-scale, and ultra-large time delay on a chip. By tailoring mode (DE)MUXes and introducing ultralow-loss multimode photonic waveguides, the TE_0_, TE_1_, and TE_2_ modes are employed to propagate in parallel in the proposed MDUs and a brand-new tunable multimode photonic delay line has been realized with the highest line delay-density of 376.9 ps/cm. For the present MDUs and 7-bit tunable multimode photonic delay line fabricated with standard MPW processes, the measured propagation losses of the TE_0_, TE_1_, and TE_2_ modes in MWSs are 0.20 dB/cm, 0.31 dB/cm, and 0.49 dB/cm, respectively. The measured overall delay loss for the MDUs is measured as low as 0.0040 dB/ps (including the loss of the mode MUXes/DEMUXes). Furthermore, the tunable multimode photonic delay line has achieved an ultra-large delay range of 12.7 ns and a delay resolution of 100 ps within an ultra-compact footprint of 3.85 mm^2^, representing the largest delay range and the highest delay density (3299 ps/mm^2^) among on-chip photonic delay lines reported to date, to the best of our knowledge.

**Fig. 4:**
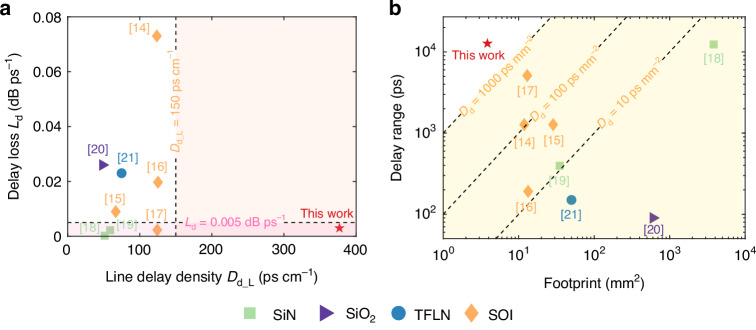
Comparison of reported on-chip tunable photonic delay lines. **a** The delay loss *L*_d_ versus the line delay-density *D*_d_L_; **b** the delay range versus the footprint

A comparison of reported on-chip tunable photonic delay lines is given in Supplementary information, Section S10. We also compare the key performance metrics in Fig. 4a, b. Figure 4a focuses on the delay loss *L*_d_ versus the line delay-density *D*_d_L_. Among the on-chip tunable photonic delay lines reported, only SiN photonic waveguides^18^ and special SOI photonic waveguide with a broadened core^17^ have the delay loss less than 0.005 dB/ps. Even though SiN photonic waveguides can potentially offer an ultra-low delay loss of 0.0002–0.0022 dB/ps^18–20^, their low group refractive index *n*_g_ (i.e., ~1.49) necessitate a long waveguide, which is 2.9× longer than that for singlemode silicon photonic waveguides with the same time dealy. Since most on-chip optical waveguides have a group refractive index less than 4.5, there is not any result reported with a line delay-density higher than 150 ps/cm. In contrast, here we introduce the ultralow-loss MDUs supporting the TE_0_, TE_1_, and TE_2_ modes propagating in parallel with the help of mode (DE)MUXes, pushing the boundary for achieving a greatly improved effective group refractive index *n*_g_ as high as ~11.3 and resulting in a record-high line delay-density of 376.9 ps/cm, which is significantly higher than the best result (125.3 ps/cm) reported previously, as shown in Fig. 4a. It is the *first* time to achieve an on-chip tunable photonic waveguide delay line with a very high line delay-density *D*_d_L_ (376.9 ps/cm) as well as a very low delay loss *L*_d_ (0.004 dB/ps).

Furthermore, Fig. 4b gives a summary for the tunable photonic delay lines developed with various photonic wavegudies in terms of the delay ranges and the footprints. For tunable SiN and SiO_2_ photonic delay lines^18–20^, their waveguides usually have a propagation loss as low as 0.01–0.1 dB/cm to enable very long delay lines. Unfortunately, their relatively large bending radii (i.e., 10^2^–10^4^ μm) prevent to achieve compact footprints, while their low thermo-optic coefficients necessitate substantial power consumption for tuning. In^18^, a tunable SiN photonic delay line was demonstrated with a time delay exceeding 10 ns but a large footprint of 3825 mm². The reported TFLN tunable photonic delay line have a large footprint as 50 mm^2^ even for a small delay range of 150 ps because it requiers a long phase arms (~2.5 mm) for on-chip electro-optic switches^21^. Therefore, the delay densities of the reported tunable SiN, SiO_2_ and TFLN photonic delay lines are difficult to exceed 10 ps/mm^2^. In contrast, tunable silicon photonic delay lines have much more compact footprints and much higher delay density up to 100 ps/mm^2^ owing to the ultra-high index-contrast Δ. However, conventional tunable singlemode silicon photonic delay lines^16^ are primarily limited in achieving long delay line due to the high propagation loss (i.e., ~2–4 dB/cm) for the waveguides fabricated by standard MPW processes. To reduce the losses, researchers have introduced shallowly-etched ridge waveguides^14^ and thin-core waveguides^15^ to extend the delay range. In particular, we had proposed high-performence tunable photonic delay lines by introducing low-loss broadened silicon photonic waveguides and compact Archimedean spiral waveguides as well as Euler S-bend^17^, enabling a large delay range of 5.11 ns and a high delay density of 393.7 ps/mm^2^_._ In this paper, our proposed tunable multimode silicon photonic delay line achieves a record-large delay range of 12.7 ns with an ultra-compact footprint of 3.85 mm^2^, leading to the record high delay density *D*_d_ of 3299 ps/mm^2^, which is an order of magnitude higher than the best one reported previously. Note that the whole tunable photonic delay line includes optical switches and input/output couplers integrated on the same chip, and consequently its delay density is lower than that of a single waveguide spiral, as given in Supplementary information, Section S3.1. Overall, the concept of developing multimode-enabled delay lines paves the way for future applications such as large aperture antennas and arrayed microwave beamforming with a high number of subarrays, OFDR and OCT for extended detection ranges, high-speed optical communication and integrated photonic quantum technologies.

## Materials and methods

### Chip fabrication method

The proposed MWSs and tunable multimode photonic delay line were fabricated at the MPW foundry (Advanced Micro Foundry Pte Ltd) with the standard processes. More details can be found in Supplementary information, Section S1.

### Simulation method

The finite-difference frequency-domain) method was utilized to calculate the mode profiles, effective refractive indices *n*_eff_, and group refractive indices *n*_g_. The transmission responses and the light propagation for the Euler S-bend and the mode (DE)MUXes were simulated with the finite-difference time-domain method. The scattering losses of the waveguide sidewalls/surfaces were calculated with the three-dimensional volume current method which is detailed in Supplementary information, Section S6.1.

### Loss measurement method

The transmission responses of the MWSs and the MDUs were measured by using an amplified spontaneous emission source and an optical spectrum analyzer. The losses of the MWSs were obtained by normalizing the transmission responses with the straight waveguide.

### Delay measurement method

The delay measurement was carried out by the phase responses of the RF single generated by vector network analyzer. The fabricated chip was wire-bonding and the MZI switches were switched by heating their micro-heaters controlled by a programmable control circuit. More details about the experimental setup of delay measurement can be found in Supplementary information, Section S8.

## Supplementary information

Supplementary information

## Data Availability

Data underlying the results presented in this paper are available from the authors upon reasonable request.
