# Supplementary material for: Multimode-enabled silicon photonic delay lines: break the delay-density limit
Source: Light Sci Appl. 2025 Mar 31;14:145. doi: 10.1038/s41377-025-01820-2 (PMC11958738; doi:10.1038/s41377-025-01820-2)
Supplement: Supplementary file 1 — Supplementary information [file 41377_2025_1820_MOESM1_ESM.docx]

Supplementary Information for: Multimode-enabled silicon photonic delay line: break the delay density limit

Shihan Hong ^1^, Long Zhang ^1^, Jiachen Wu ^1^, Yingying Peng ^1^, Linyan Lyu ^1^, Yinpeng Hu ^1^, Yiwei Xie ^1^ and Daoxin Dai ^1,2,3^*

1. State Key Laboratory for Extreme Photonics and Instrumentation, College of Optical Science and Engineering, International Research Center for Advanced Photonics, Zhejiang University, Zijingang Campus, Hangzhou 310058, China.

2. Intelligent Optics and Photonics Research Center, Jiaxing Research Institute, Zhejiang University, Jiaxing 314000, China.

3. Ningbo Research Institute, Zhejiang University, Ningbo 315100, China.

* Corresponding author: [dxdai@zju.edu.cn](mailto:dxdai@zju.edu.cn).

**Contents**

[S1. Fabrication method 3](#_Toc183683693)

[S2. Two tunable photonic delay line schemes 4](#_Toc183683694)

[S3. Additional information for performants of multimode waveguide spiral and delay line 5](#_Toc183683695)

[**S3.1. Delay density comparison of single waveguide spiral for different waveguide structures/platforms** 5](#_Toc183683696)

[**S3.2. Delay bandwidth of the multimode delay line** 6](#_Toc183683697)

[**S3.3. Discussion on the extensibility of the higher-order modes** 6](#_Toc183683698)

[S4. Additional information for design and characterization of the discrete devices 8](#_Toc183683699)

[**S4.1. Design of low-loss and compact multimode Euler S-bend** 8](#_Toc183683700)

[**S4.2. Design and characterization of low-loss mode (DE)MUXes** 8](#_Toc183683701)

[**S4.3. Optimized phase-error and switch speed of optical switch** 10](#_Toc183683702)

[S5. Light propagation order of three guided-modes in the MDU 12](#_Toc183683703)

[S6. Additional information for scattering losses analysis 13](#_Toc183683704)

[**S6.1. Three-dimensional volume current method** 13](#_Toc183683705)

[**S6.2. Analysis of the scattering losses for different polarizations and modes** 13](#_Toc183683706)

[S7. Additional results for data transmission in the MWS and the MDU 16](#_Toc183683707)

[S8. Experimental setup for the delay measurement 17](#_Toc183683708)

[S9. The overall transmission for tunable multimode photonic delay line 18](#_Toc183683709)

[S10. Performance comparison of on-chip tunable photonic delay lines 19](#_Toc183683710)

[References 20](#_Toc183683711)

**S1. Fabrication method**

In this work, all the designed devices were fabricated at Advanced Micro Foundry Pte Ltd. based on the silicon-on-insulator (SOI) platform with a 220-nm thick silicon top layer and a 3-μm thick oxide buried layer. A 3-μm thick oxide cladding was deposited onto the fabricated silicon waveguides. The 120-nm thick titanium-nitride (TiN) alloy and 2-μm thick aluminum (Al) thin films were deposited and patterned to realize high-resistance heaters and low-resistance routing wires, respectively.

**S2. Two tunable** **photonic delay line schemes**

Here, we provide a detailed explanation of the functional implementation and delay characteristics of two tunable photonic delay line structures, as shown in Fig. S1. The first scheme is on-chip resonant delay line structures, via, e.g., microring resonators (MRRs), cascaded MRRs, Bragg gratings and photonic-crystal waveguides, as shown in Fig. S1(a). From the typical relationship between delay and wavelength shown in the middle, resonant tunable delay lines generally achieve delays surpassing the waveguide length at resonant wavelengths. However, the bandwidth is limited by the time delay since the delay-bandwidth product is constant. Take all-pass MRR as an example, the delay bandwidth product is 2∕π ^1^. Its bandwidth is only 0.64 GHz for a 1 ns time delay, which is far from sufficient for large-aperture, large-scale beamforming systems. Cascaded MMRs can increase bandwidth by stitching together resonance peaks, but this requires precise control of each microring and can introduce significant delay errors at the stitching boundaries.

The second scheme achieves a digital tunable delay by routing the light propagation to go through the waveguide delay lines with different physical lengths with the assistance of on-chip optical switches (OS) in cascade, as shown in Fig. S1(b). Since the group refractive index *n*_g_ of an optical waveguide changes minimally within a certain wavelength range, the delay is essentially wavelength-insensitive, as the delay spectrum shown in top right region of Fig. S1(b). The physical-length delay lines primarily achieve high-density arrangement through spiral waveguide and serpentine waveguide structures. Although serpentine waveguide can flexibly change their arrangement direction according to the delay line layout, the need to redirect the straight waveguide through multiple bend waveguide accumulates mode mismatches, further increasing waveguide losses. For spiral waveguide, the curvature-gradient waveguide structure can avoid mode mismatches during propagation. Additionally, due to the curvature mismatch between adjacent waveguides spiral, spiral waveguide allows for denser waveguide arrangements, thereby achieving higher delay density. Hence, we use waveguide spiral structure to construct our multimode waveguide spiral (MWS), multimode delay unit (MDU) and the corresponding tunable photonic delay line.


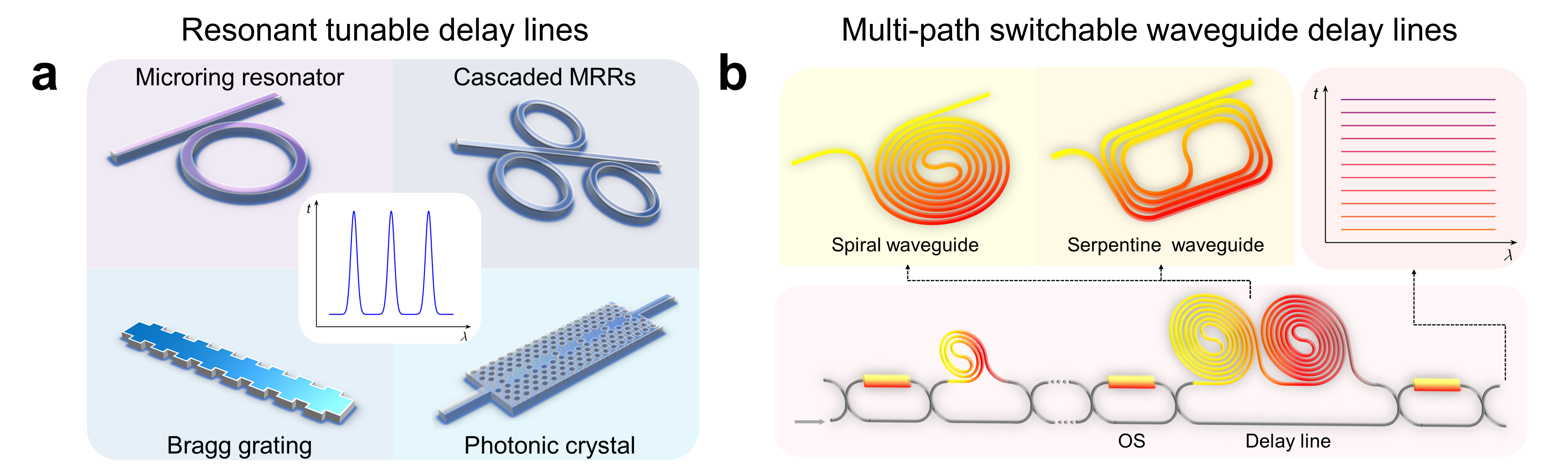


**Figure S1 | Concept of two tunable delay line schemes. a** Schematic of tunable photonic delay line based on resonant dispersive devices as microring resonators (MRRs), cascade MMRs, Bragg gratings and photonic-crystal waveguides. Middle region is their typical delay spectrum. **b** Schematic of tunable photonic delay line based on multi-path switchable delay line with spiral or snake-like waveguide. Top right region is their typical delay spectrum.

**S3. Additional information for performants of multimode waveguide spiral and delay line**

## **S3.1. Delay density comparison of single waveguide spiral for different waveguide structures/platforms**

In this work, we introduce a figure of merit defined as the delay density *D*_d_, which is given as the time delay per unit area (ps mm^-^²), to characterize the time-delay capability and the footprint compactness for an optical delay line. Here, we employ single waveguide spirals as delay unit to calculate the relationship between time delay and device footprint across different waveguide structures/platforms for the reasons given in Supporting Information, Section 2. Here, we compare the delay density of single waveguide spirals for the different waveguide structures/platforms from the known standard processes and published works, i.e., SOI- multimode and singlemode waveguide ^2^, SOI - thin waveguide ^3^, SOI - ridge waveguide ^4^, thin film lithium niobate waveguide ^5^ and silicon nitride waveguide ^6^. The time delays of single waveguide spirals are simulated based on the group refractive index *n*_g_, bend radii, waveguide gap *w*_g_, and delay loss *L*_d_ mentioned in the references. Here, the silicon nitride waveguide in ^7^ and silica waveguide in ^8^ are not considered because their large footprint results in delay densities significantly lower compared to other works. For ease of comparison and to meet practical application requirements, we set the upper limit of waveguide loss at 30 dB for all waveguide spirals to calculated the corresponding waveguide lengths and subsequently obtained their upper delay limits.


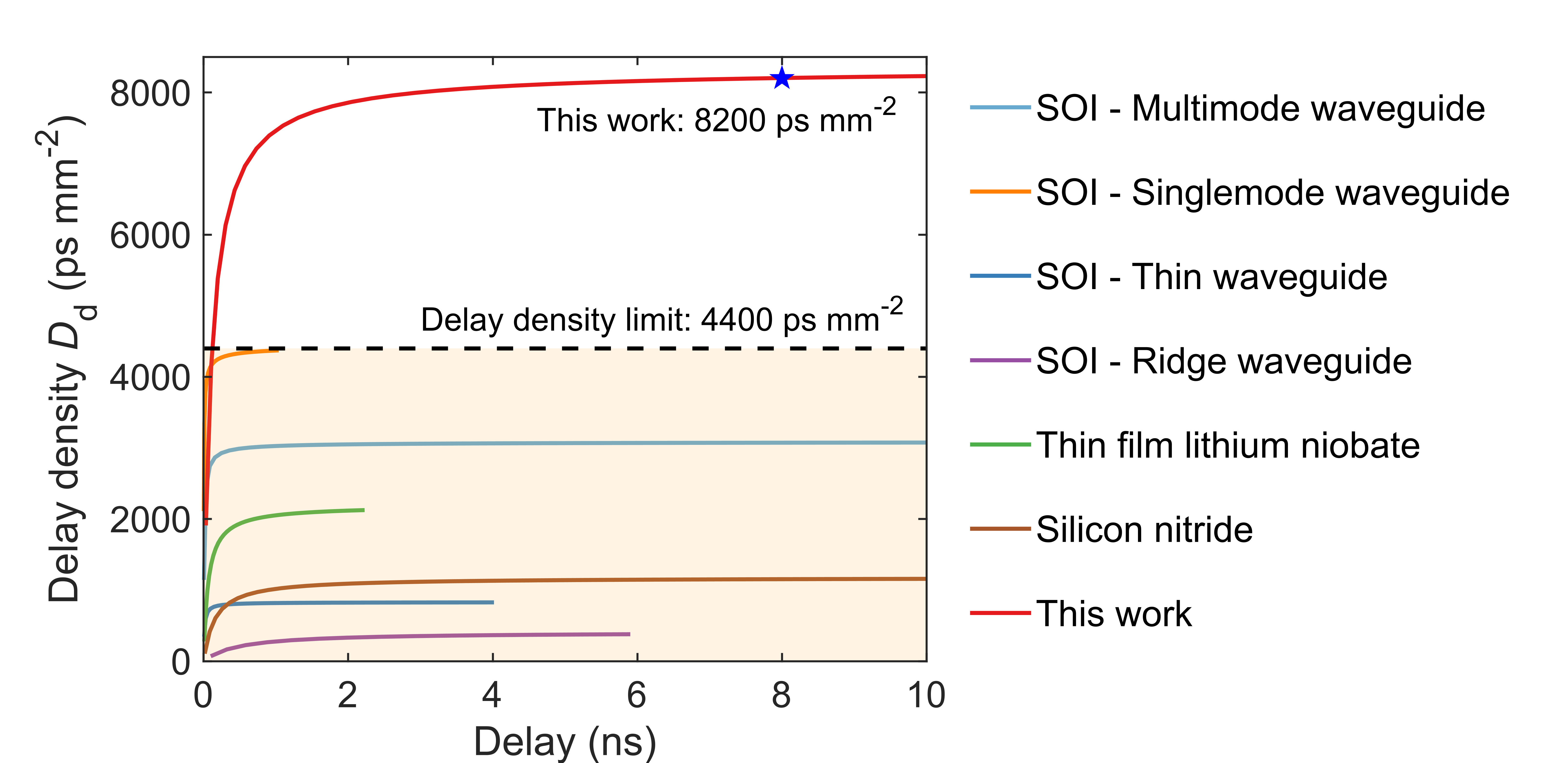


**Figure S2 |** **Delay density versus delay of single waveguide spirals for different waveguide** **structures/platforms.**

The results of delay density versus time delay of single waveguide spirals for different waveguide structures/platforms are shown in Fig. S2. For all type of waveguide structures/platforms, the delay density gradually increases when the time delay is relatively small, and then remains a constant value. This is because the central S-bend occupies a relatively large footprint when the spiral radius is small, resulting in the lowest delay density. As the time delay increases, the increase in delay and footprint tends to become linear, thus the delay density approaches a constant value. At this point, the delay density is mainly determined by the group refractive index *n*_g_ and the waveguide gap *w*_g_. Given that the currently proposed SOI-multimode waveguide spiral ^2^ and silicon nitride waveguide spiral ^6^ exhibit low propagation losses (0.28 dB cm^-1^, 0.13 dB cm^-1^), they can achieve on-chip time delay higher than 10 ns. However, due to their relatively small group refractive index *n*_g_, their delay density *D*_d_ is less than 3000 ps mm^-2^. The SOI-singlemode waveguide spiral can reach the delay density limit to 4400 ps mm^-2^ in the state-of-art structures, but the unacceptable propagation loss prevents it to achiever a time delay over 1 ns. Our proposed MWS, due to the low propagation loss and high effective group index of the broadened multimode waveguides, not only achieves time delay higher than 10 ns within limited losses but also surpasses the current delay density limits, reaching an ultra-high delay density of 8200 ps mm^-2^. It is worth noting that the footprint of a tunable photonic delay line also includes devices such as optical switches, referenced waveguides, and input/output couplers, as well as the design layout. These factors result in the actual delay density of the tunable photonic delay line being lower than that of theoretical simulation result for single waveguide spiral.

## **S3.2.** **Delay bandwidth of the multimode delay line**

Note that the elements used for the MWS (including the multimode Euler S-bends, (DE)MUXes and optical switches) are designed to be broadband (over e.g., 60 nm). Furthermore, the waveguide’s group refractive indices are also wavelength-insensitive, particularly for the real applications when the wavelength range considered is small. For example, for microwave photonics with a typical microwave frequency range of < 40 GHz, the variations of the group indices *n*_g_TE0_, *n*_g_TE1_ and *n*_g_TE2_ are 0.00012, 0.00009, and 0.00006, respectively, resulting a small delay variation of only 0.238 ps for a long delay of 10 ns.

## **S3.3.** **Discussion on the extensibility of the higher-order modes**

To further investigate the feasibility of extending higher-order modes in the present multimode photonic waveguide delay line, we analyze the delay performance of the TE_0_ - TE_10_ modes for different waveguide widths, as shown in Figure S3.

As it can be seen, when the waveguide width is relatively small (e.g., 3, 4 μm), those higher-order modes still experience notable scattering losses due to the substantial portion of their mode field intensity at the waveguide sidewalls/surfaces. When increasing the waveguide width to more than e.g., 6 μm, the group refractive indices and the scattering losses becomes insensitive to the mode order, showing that the TE_0_-TE_10_ modes have similar group refractive indices and scattering losses in theory. We also calculate the line delay-density *D*_d_L_ and the delay loss *L*_d_ when working with different mode numbers, as shown in Figures S3(c)-S3(d). Here the core width is varied from 3 μm to 10 μm. From those figures, one sees that the line delay-density *D*_d_L_ increases with the mode number as expected, while the delay loss *L*_d_ converges to ~ 0.0026 dB ps^-1^ when the core width exceeds 6 μm. This is because all modes in an ultra-wide strip waveguide approximate to the slab modes in a planar waveguide, thus exhibiting similar group refractive indices and scattering losses. Consequently, when the MWS simultaneously propagates the TE_0_ - TE_10_ modes, the line delay-density *D*_d_L_ reaches as high as 1300 ps cm^-1^. Therefore, one can optimally choose the core width of as 6 μm for achieve a high delay density with the minimized loss and the footprint.


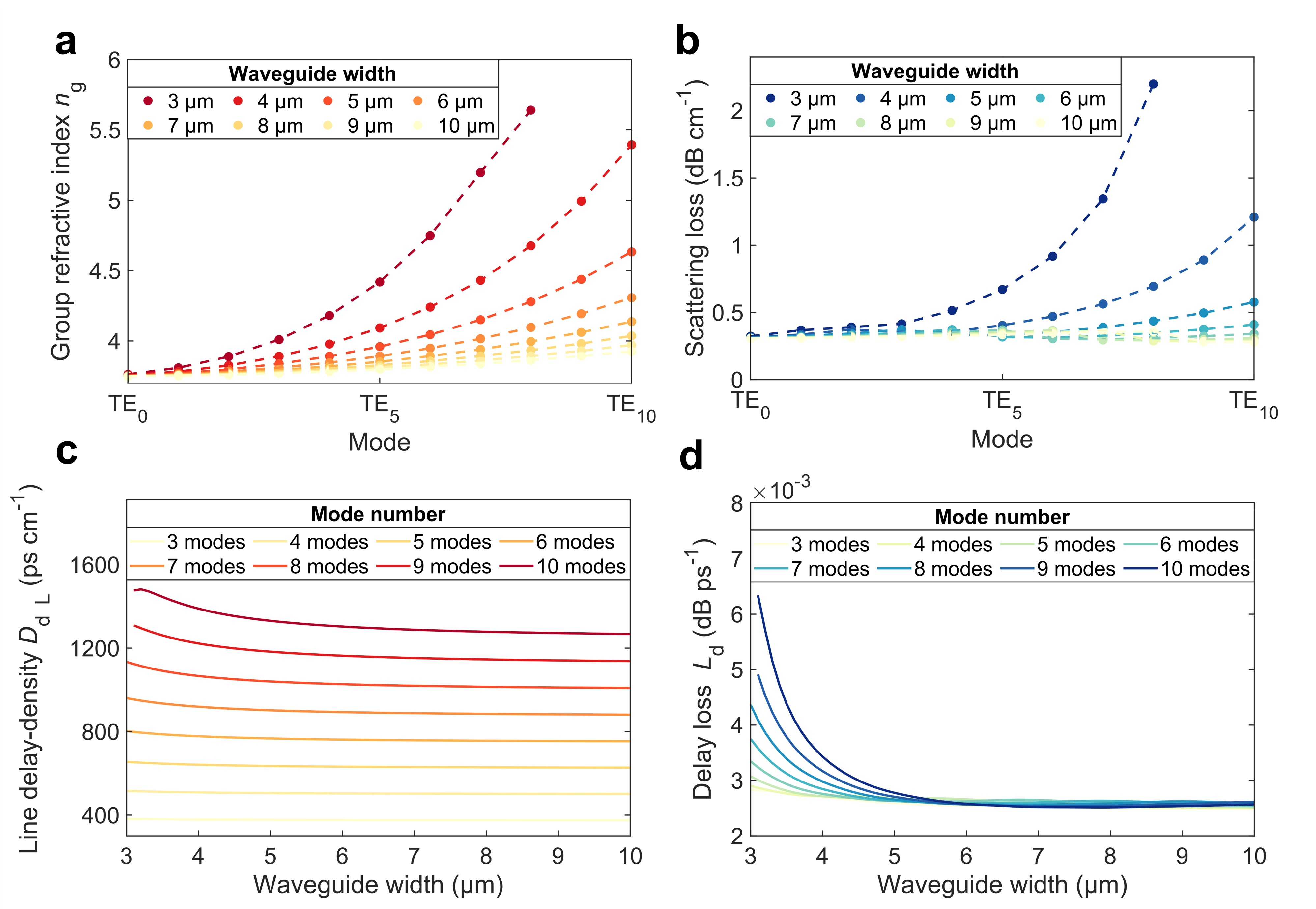


**Figure S3 |** **Analysis of the delay performances for higher-order modes and broader waveguide.** **a** Calculated group refractive indices *n*_g_ and **b** scattering losses for the TE_0_-TE_10_ modes at different waveguide width *W*_wg_. **c** Calculated line delay-density *D*_d_L_ and **d** delay loss *L*_d_ with different mode number at various waveguide width *W*_wg_.

One should note that the practical limit of the mode number lies in the mode MUXes/DEMUXes. In principle, the mode MUXes/DEMUXes with many mode-channels can be easily achieved by utilizing the adiabatic directional couplers or even asymmetric directional couplers ^9–11^. However, the mode MUXes/DEMUXes working with more than ten mode channels have not been demonstrated with high performances yet due to the fabrication errors. More efforts should be made to achieve high-performance multimode photonic devices with more mode-channels in the future.

**S4. Additional information for design and characterization of the discrete devices**

## **S4.1.** **Design of low-loss and compact multimode Euler S-bend**

In order to ensure ultra-compact size and ultralow bending losses as well as negligible higher-order-mode excitation for three guided-modes in the MWS, we employ the Euler S-bend in the middle of the MWS to avoid an abrupt change of the bending radii from *R* to −*R*. More design details can be find in Ref ^12^. The main challenge here is to design low-loss bends for three guided-modes. Here, the curvature radii *R*_max_ and *R*_min_ for the Euler S-bend are chosen as 60 μm and 24 μm, respectively, and the *W*_out_ and *W*_in_ are set as 3 μm and 1.2 μm. Figures S4(a)-S4(c) show the simulated light propagation of the designed Euler S-bend for the TE_0_, TE_1_ and TE_2_ modes, respectively. It can be seen that the launched modes propagate smoothly along Euler S-bend, and no multimode interferences are observed, indicating that the intermode crosstalks are low. The corresponding calculated mode-excitation ratios monitored at the output port are shown in Figs. S4(d)-S4(f). It can be observed that the excess losses for three guided-modes in the Euler S-bend are negligible, and the internal mode crosstalks are smaller than −20 dB in the wavelength range of 1500 - 1600 nm. Therefore, the proposed Euler S-bend works very well with low-loss and low-crosstalk light propagation of the three guided-modes even when the footprint is as compact as 120 × 80 μm. Such design technique plays a crucial role in large-scale signal processor with numerous elements.


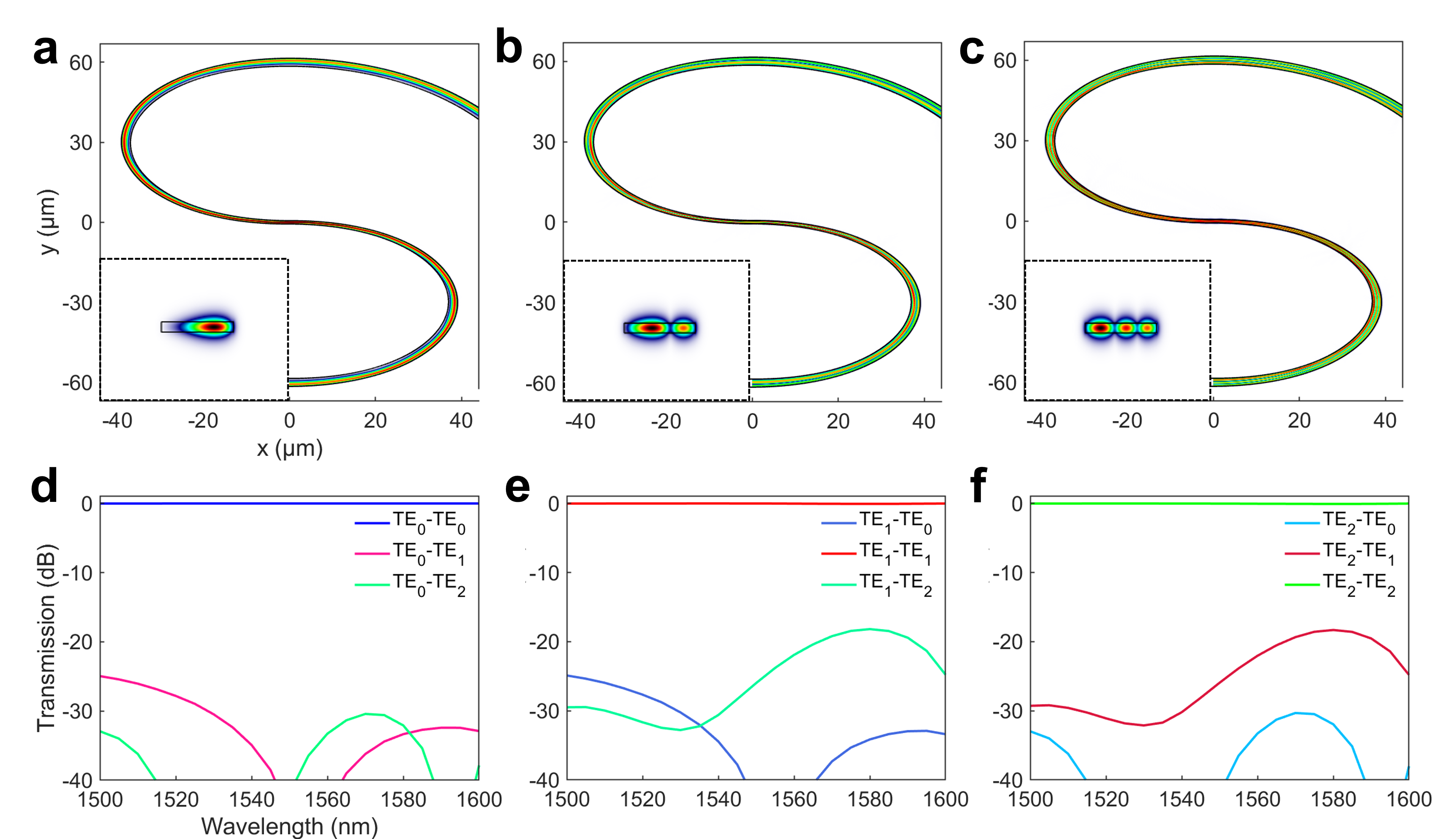


**Figure S4 |** **Euler S-bend for three guided-modes.** **a-c** Simulated light propagation of the TE_0_, TE_1_ and TE_2_ mode in the Euler S-bend. **d-f** The corresponding calculated excess loss and intermode crosstalk.

## **S4.2.** **Design and characterization of low-loss** **mode** **(DE)MUXes**

In order to ensure low-loss mode conversion, we choose the adiabatic direction couplers (ADCs) as the mode (DE)MUXes ^9,10,13–15^. By careful design the parameters of access/bus waveguides to the mode match regime, the mode (DE)MUXes can achieve a broadband and low-loss (de)multiplexing for three guided-modes. More details can be found in Refs ^9,10,15^. The parameters of the designed mode (DE)MUXes for two higher-order modes are shown in Table. S1 and the simulated results are demonstrated in Fig. S5. The propagation profiles of the TE_1_ and TE_2_ mode (DE)MUXes (shown as Figs. S5(a) and S5(b)) reveal that the input TE_1_ and TE_2_ modes in the bus waveguide can be totally converted into the TE_0_ mode in the access waveguide. The corresponding calculated transmissions are shown in Figs. S5(c) and S6(d), reveal that the excess losses < 0.02 dB and crosstalk < -40 dB for both mode (DE)MUXes.

Table S1. Parameters of the designed mode (DE)MUXes for TE_1_ and TE_2_ modes

| Parameters (μm) | Gap | *L*_c_ | *W*_a1_ | *W*_a2_ | *W_b_*_1_ | *W*_b2_ |
| --- | --- | --- | --- | --- | --- | --- |
| TE_1_ | 0.2 | 40 | 0.18 | 0.28 | 0.74 | 0.5 |
| TE_2_ | 0.2 | 50 | 0.26 | 0.32 | 1.04 | 0.93 |


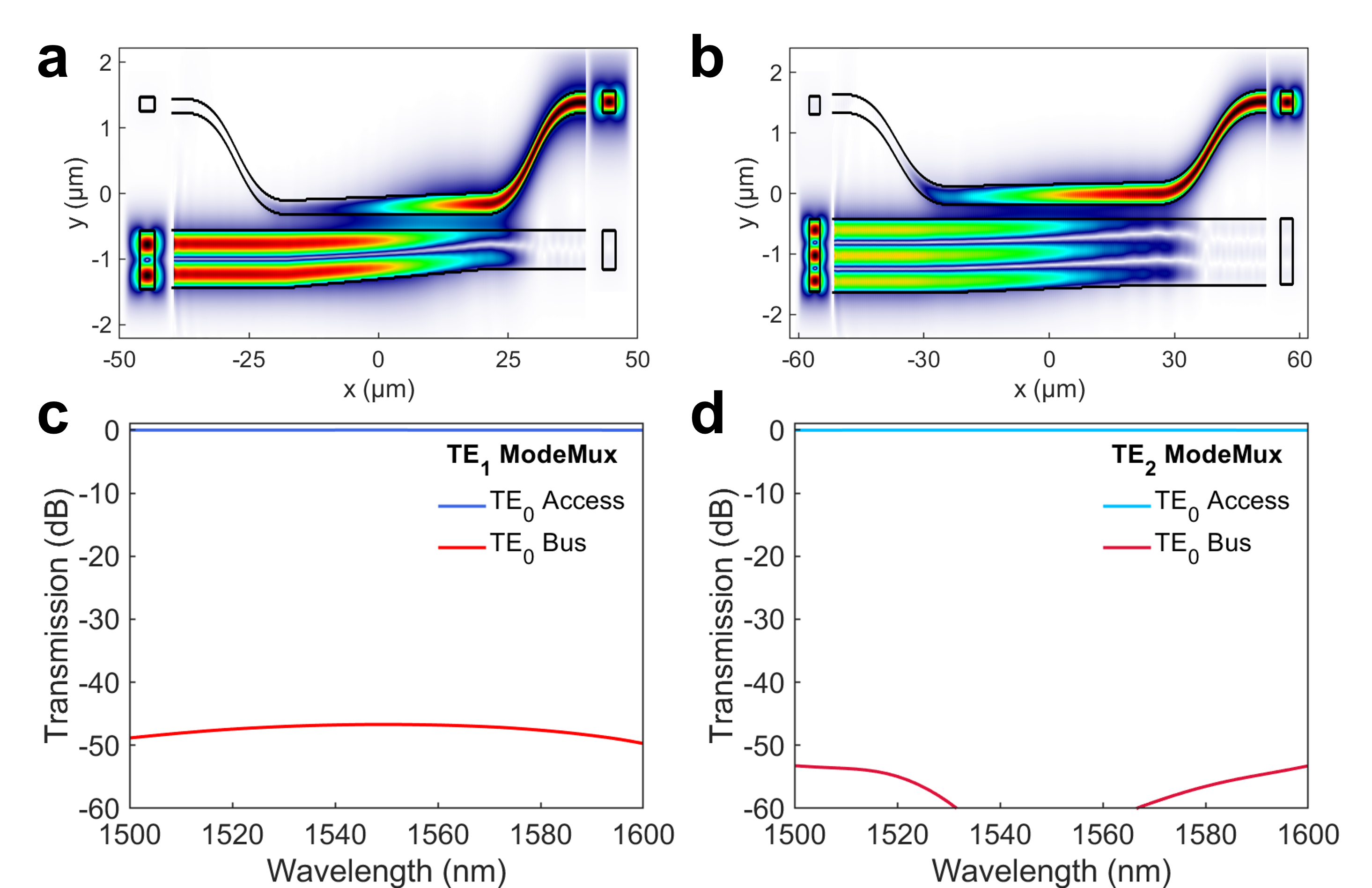


**Figure S5 |** **Simulation results for mode (DE)MUXes.** Simulated light propagation of the **a** TE_1_ and **b** TE_2_ mode (DE)MUXes. **c-d** The corresponding calculated transmissions.

The fabricated structure is a pair of mirrored mode MUXes linked by a straight multimode waveguide, as shown in Fig. S6(a). The TE_0_ mode can be directly launched, while TE_1_ and TE_2_ can be excited by respective mode (DE)MUXes marked in green and red region. The measure results are normalized by the reference straight waveguide. The measured transmittance spectra are shown in Figs. S6(b)-S6(d). As shown experimentally, the excess losses of the mode MUXes/DEMUXes are respectively ~0.15 dB, 0.45-1.1 dB, 0.3-0.4 dB for the TE_0_, TE_1_, and TE_2_ modes, while the measured crosstalk is below −20 dB, in the wavelength range of 1520-1580 nm. The TE_1_ mode (DE)MUX shows a higher loss mainly due to the mode hybridness of TE_1_ and TM_0_ cause by the tilted waveguide sidewalls (tilt angle ≈ 84°). Subsequent steps can involve optimizing the mode (DE)MUXes width variations to avoid the hybridization region, thereby reducing device losses.


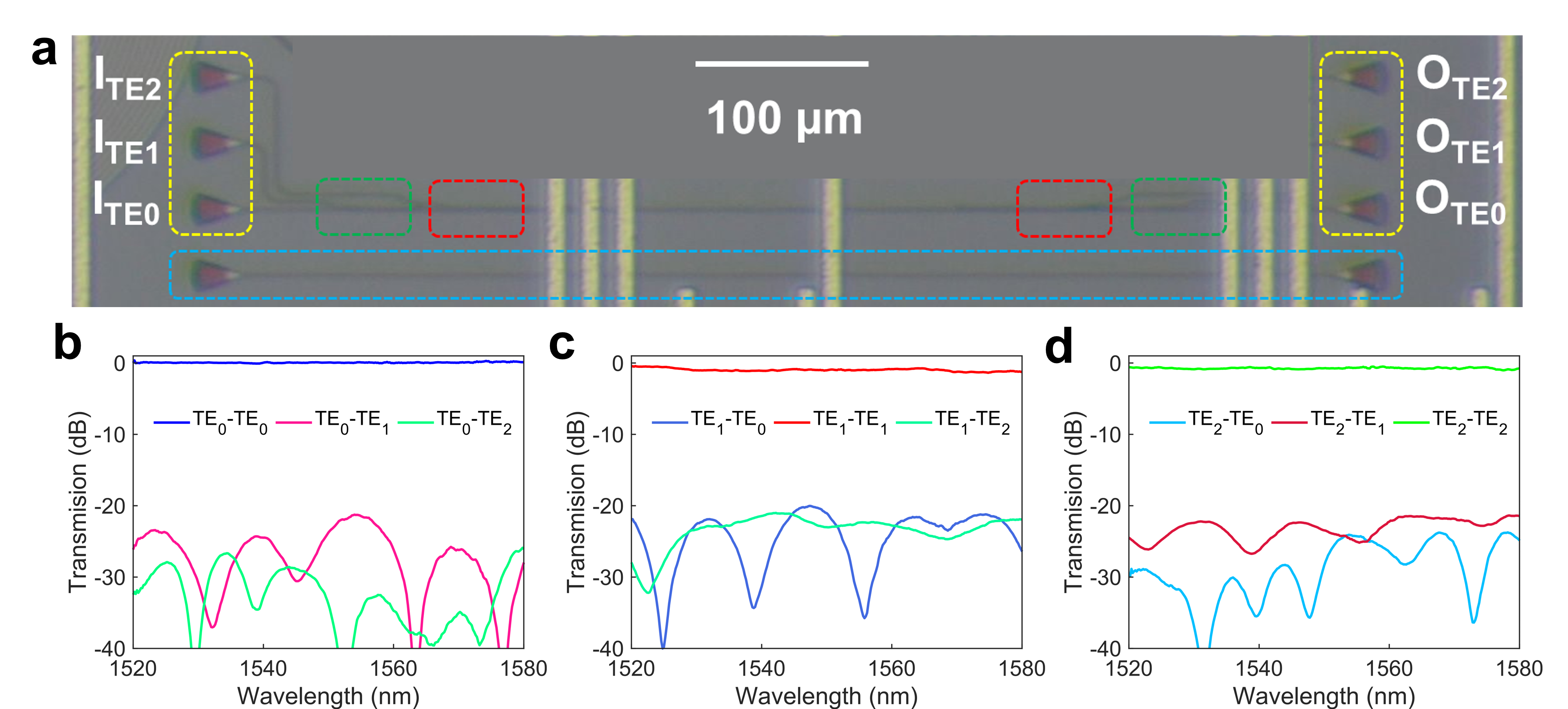


**Figure S6 |** **Measurement results for** **mode** **(DE)MUXes.** **a** The optical microscope image of fabricated devices with port identifiers labeled. (Yellow region: grating coupler; green region: TE_1_ mode (DE)MUX; red region: TE_2_ mode (DE)MUX; blue region: reference straight waveguide.) **b-d** The measured transmittance spectra for TE_0_-TE_2_ mode (DE)MUXes.

## **S4.3.** **Optimized phase-error and switch speed of optical switch**

Our optical switch consists of two 2×2 3-dB multimode interference (MMI) couplers and two symmetric Mach–Zehnder interferometer (MZI) arms, as shown in the device schematic in Fig. S7(a). Traditional MZIs face phase errors challenges due to waveguide width changes during fabrication. Here we introduce a low-phase-error MZI by broadening the core-width of the calibration-free phase shifters (CFPSs) to be 2 μm, significantly reducing random phase errors compared to conventional MZIs with 0.45-μm-wide singlemode phase shifters. For thermo-optic modulated optical switches, the main limitation on switching speed is determined by the falling edge speed of the optical switch, which is related to the material's heat dissipation performance. To enhance the heat dissipation speed of the CFPS in the optical switch, we have introduced silicon slabs at both ends of the phase shifter to increase its thermal conductivity. The length of the CFPS is set as 80 μm. The spacing between the CFPS and the slabs is set to 0.3μm, ensuring that it is kept as close to the CFPS as possible without inducing coupling. The optical microscope image of fabricated device is shown as Fig. S7(b). The fabricated MZI switches (Fig. S7(c)) exhibit low excess losses below 0.5 dB and high extinction ratios higher than 25 dB in the wavelength range of 1520-1580 nm. The simulated and measured switching speed is shown as Fig. S7(d), demonstrating that the optical switch has a rise/fall time of 13.47/14 μs in experiment, which are similar to the simulation results of 11.7/11.8 μs respectively for rise/fall time.


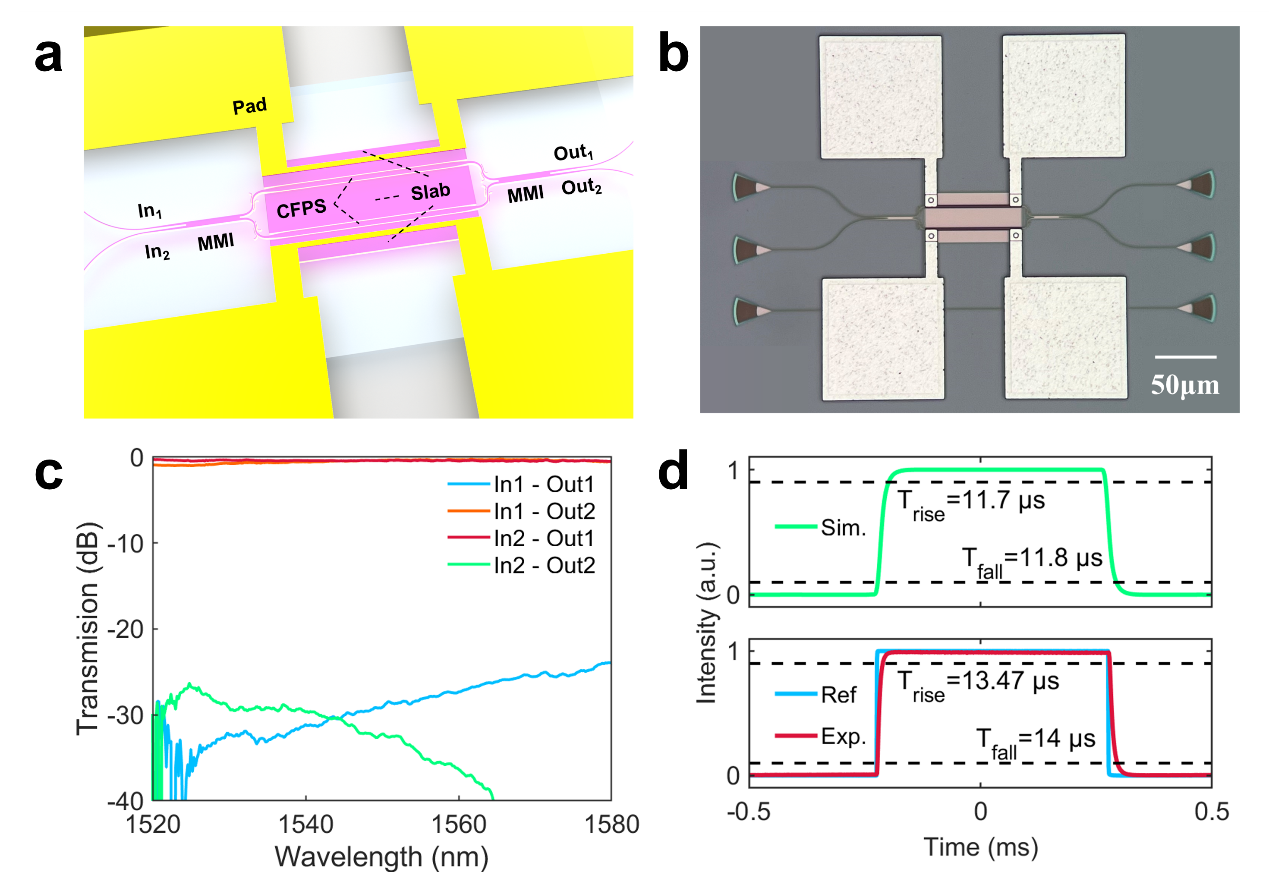


**Figure S7 |** **Optimized switch speed MZI with slab design.** **a** 3D schematic of the optical switch. **b** The optical microscope image of fabricated device. **c** The measured transmittance spectra. **d** Simulated and measured switching speed.

**S5.** **Light propagation order of three guided-modes in the MDU**

To explain the light propagation order more accurately in the MDU, we illustrate the schematic diagram of propagation order as shown in Figure S8. The illustrative diagram of structures in the MUD are shown in Fig. S8(a), which consists of, from left to right: the input waveguide, TE_1_ mode MUX, TE_2_ mode MUX, MWS, TE_2_ mode DEMUX, TE_1_ mode DEMUX and the output waveguide. The TE_1_ mode MUX and TE_1_ mode DEMUX, TE_2_ mode MUX and TE_2_ mode DEMUX are connected by TE_0_-TE_1_ loop waveguide and TE_0_-TE_2_ loop waveguide, respectively.

As shown in Figs. S8(b)-S8(d), within the MUD, three guided-modes propagate in the following way:

(1) The input TE_0_ mode ① passes through the TE_1_ and TE_2_ mode MUXes ② with negligible loss and enters the low-loss MWS ③. Upon exiting from the spiral ④, the TE_0_ mode passes through the TE_2_ and TE_1_ mode DEMUXes ⑤ with very low loss, then goes through the corresponding TE_0_-TE_1_ loop waveguide back to the access waveguide of the TE_1_ mode MUX ⑥.

(2) The TE_0_ mode is converted to the TE_1_ mode ⑦ at the bus waveguide by the TE_1_ mode MUX. The TE_1_ mode then goes through TE_2_ mode MUX ⑧, MWS ⑨ and TE_2_ mode DEMUX ⑩. At the TE_1_ mode DEMUX, the TE_1_ mode is converted to the TE_0_ mode ⑪ into the TE_0_-TE_2_ loop waveguide and goes back to the access waveguide of the TE_2_ mode MUX ⑫.

(3) The TE_0_ mode is converted to the TE_2_ mode ⑬ at the bus waveguide by the TE_2_ mode MUX and it also goes through the MWS ⑭ and is then converted to the TE_0_ mode ⑮ by the TE_2_ mode DEMUX. Finally, the TE_0_ mode exits from the output port ⑯.

In this way, light triply goes through the same waveguide spiral with different guided-modes, accordingly enhancing the effective group index *n*_g_ of this waveguide spiral to the sum of *n*_g_TE0_, *n*_g_TE1_ and *n*_g_TE2_, and significantly increasing the total delay time.


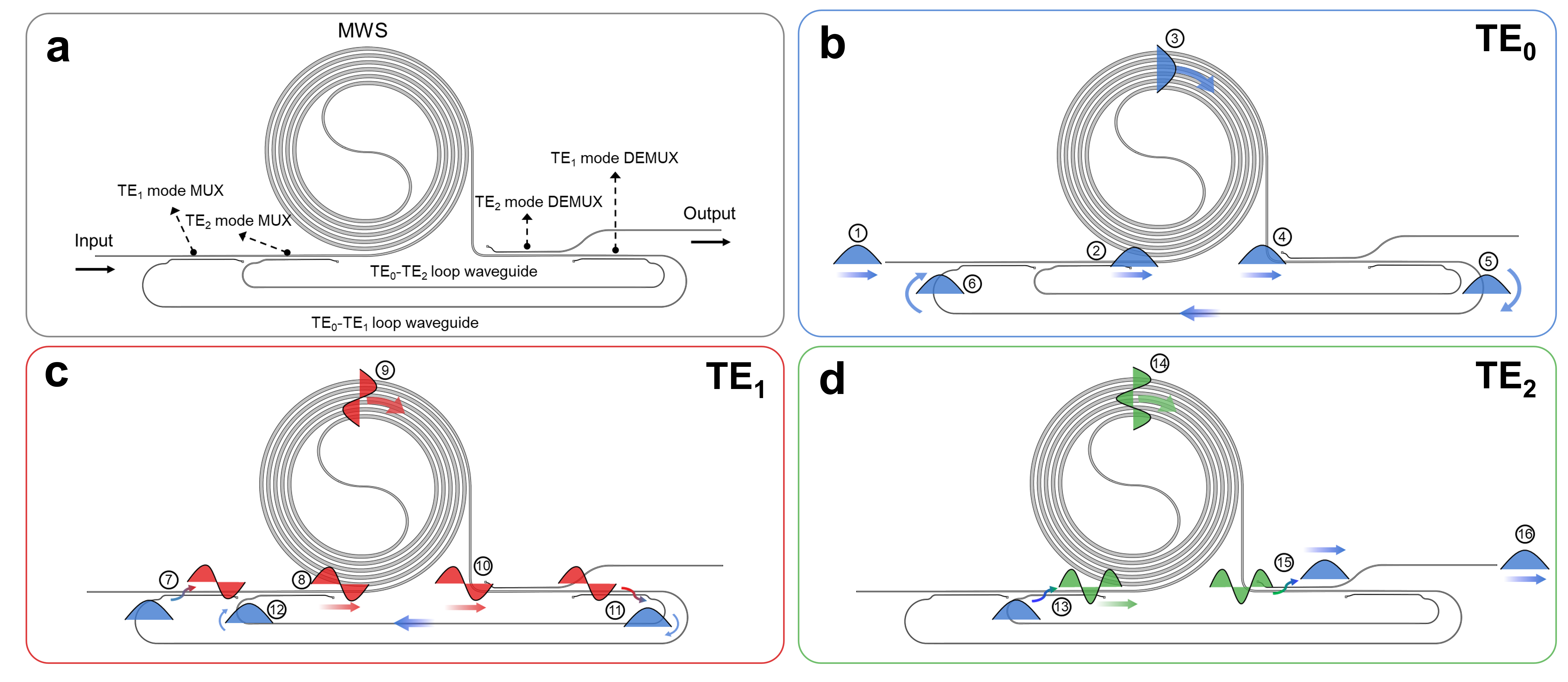


**Figure S8 | Schematic diagram of** **light propagation order in MDU. a** Structures of the MDU. **b-d** Light propagation of the TE_0_, TE_1_ and TE_2_ modes. Each mode propagates in the way indicated by the labeled numbers.

**S6. Additional information for** **scattering losses** **analysis**

## **S6.1. Three-dimensional volume current method**

The loss of on-chip waveguides is influenced by various factors, including material absorption, Rayleigh scattering, interfacial scattering, substrate leakage, bend radiation, and so on. With process optimization and careful design, many losses can be effectively reduced and mitigated (<0.01 dB/cm) ^16^. In the standard MPW processes, the majority source of the waveguide loss is scattering loss resulting from the waveguide interfacial roughness, including surface and sidewall roughness. The three-dimensional schematic of the waveguide surface/sidewall roughness is presented in Fig. S9(a), with Fig. S9(b) illustrating roughness profile, *f*(z) along the *z* axis, respectively, gives the deviation of the core-cladding interface from its mean location. The correlation length of the sidewall and surface roughness are defined as *L*_c_sidewall_ and *L*_c_surface_, respectively. Notably, the sidewall roughness *σ*_sidewall_ (*~* 1-10 nm) is much larger than surface roughness *σ*_surface_ (~ 0.3 nm). In traditional singlemode waveguides, the scattering losses induced by the sidewalls are much higher than those induced by the top/bottom surfaces, hence the scattering losses from the top/bottom surfaces are often neglected. However, as the increasing of the waveguide width, losses induced by the sidewalls decrease exponentially. When the width is sufficiently large, losses from the top/bottom surfaces become dominant. Here, we introduce the three-dimensional volume current method ^12,17,18^ by modeling the radiation loss due to scattering from the refractive index inhomogeneity at this rough interface as an equivalent polarization volume current density. This equivalent source is proportional to the electric field at the core-cladding interface of the waveguide.


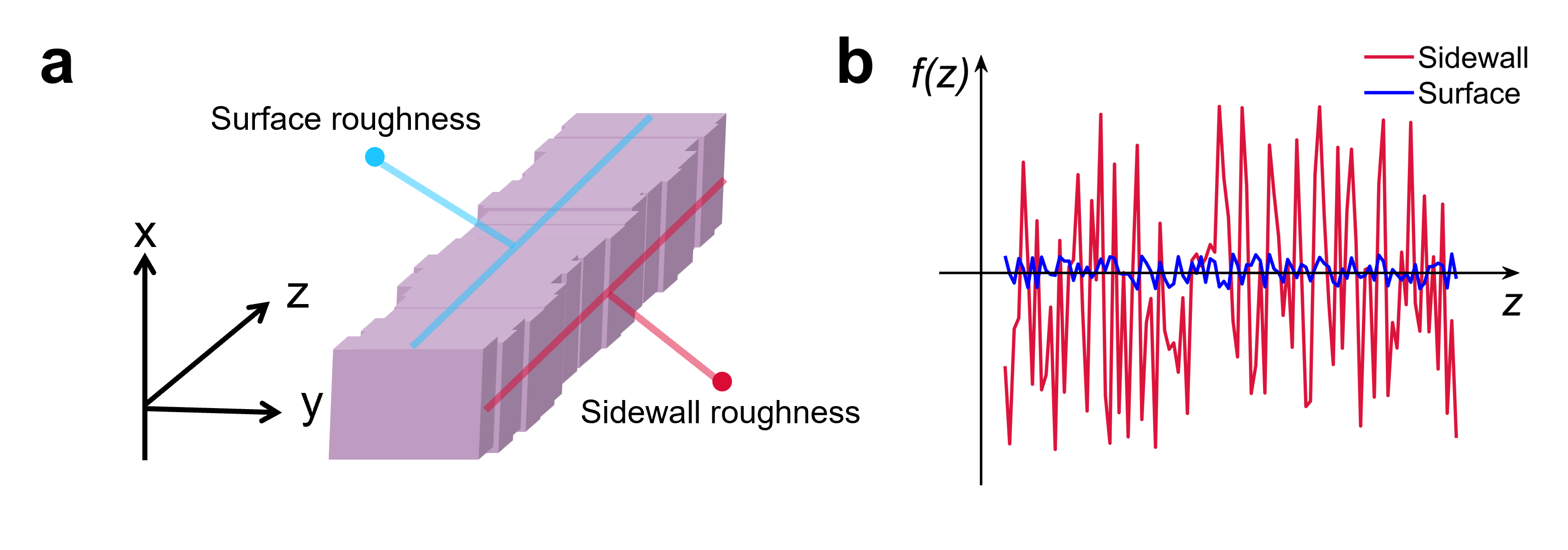


**Figure S9 |** **Three-dimensional volume current method.** **a** The schematic configurations of waveguide surfaces/sidewalls. **b** Waveguide roughness profiles for the sidewalls and the top-surface.

**S6.2. Analysis of the scattering losses for different polarizations and modes**

The selection of polarizations and modes is the fundamental for ensuring low-loss propagation in multimode delay lines. The scattering losses at the sidewalls and top/bottom surfaces of the waveguide for different guided-modes are quantified by using three-dimensional volume current method mentioned in Supplementary information, Section S6. Here the TE_0_ and TM_0_ modes are taken as examples to analyze the propagation losses of two polarization modes. The calculated mode profiles of TE_0_ and TM_0_ mode at singlemode (*W*_wg_ = 0.45 μm) and multimode waveguide (*W*_wg_ = 3 μm) are shown in Fig. S10(a). For a singlemode waveguide, there is field enhancement at the sidewalls for the TE_0_ mode, while the TM_0_ mode has field enhancement at the top/bottom surfaces. For standard MPW processes, the sidewall roughness *σ*_sidewall_ is 1-10 nm due to a dry etching, which is typically an order of magnitude higher than that for the top/bottom surface (*σ*_surface_ ~ 0.3 nm) cause by polished/deposited thin films. Consequently, the sidewalls’ scattering loss is dominant for singlemode silicon photonic waveguides, resulting in the scattering losses difference in sidewalls (which TE_0_ mode is higher than that TM_0_ mode) is higher than that top/bottom surfaces (which TM_0_ mode is higher than that TE_0_ mode), as shown in Figs. S10(b)-S11(c). Therefore, the total propagation loss for the TM_0_ mode (~ 1.2 dB cm^-1^) is lower than the TE_0_ mode (~ 2 dB cm^-1^) in experiments, which is consistent with our simulation results marked by dotted line in Fig. S10(d). Therefore, in some integrated silicon photonic devices, the TM_0_ mode often take the place of the TE_0_ mode in singlemode waveguide to reduce propagation loss. As the core width increases, the mode field intensity at the sidewalls can be reduced greatly for both polarization modes, while the scattering loss from the top/bottom surface becomes predominant. As a result, the TM_0_ mode has much higher scattering losses than the TE_0_ mode due to much stronger filed intensity at the top/bottom surface for a broadened waveguide, as shown in Fig. S10(c). Finally, as shown in Fig. S10(d), the TM_0_ and TE_0_ modes have a total loss of 0.6 dB cm^-1^ and 0.1 dB cm^-1^ when the waveguide width is broadened to 3 μm in theory. The calculated scattering loss results for TM_1_ mode are also revealed and it can be seen that the loss trend of TM_1_ mode is similar to that of TM_0_ mode. When the waveguide width exceeds 3 μm, its loss remains steady and is several times higher than that of TE_0_ mode. It demonstrates that our result also applies to higher-order TM-polarization modes. Furthermore, TM-polarization modes are prone to mode hybridization ^10^ due to non-vertical sidewalls, leading to additional mode coupling loss and inter-mode crosstalk. Therefore, the fundamental and higher-order modes of TE-polarization are considered in this paper. It can be seen from Fig. S10(d) that the loss trend for TE, TE_1_ and TE_2_ modes remains consistent with changes in width and reduced to a relatively low propagation loss of *L*_TE0_ = 0.14 dB cm^-1^, *L*_TE1_ = 0.21 dB cm^-1^, *L*_TE3_ = 0.34 dB cm^-1^ at a waveguide width *W*_wg_ = 3μm


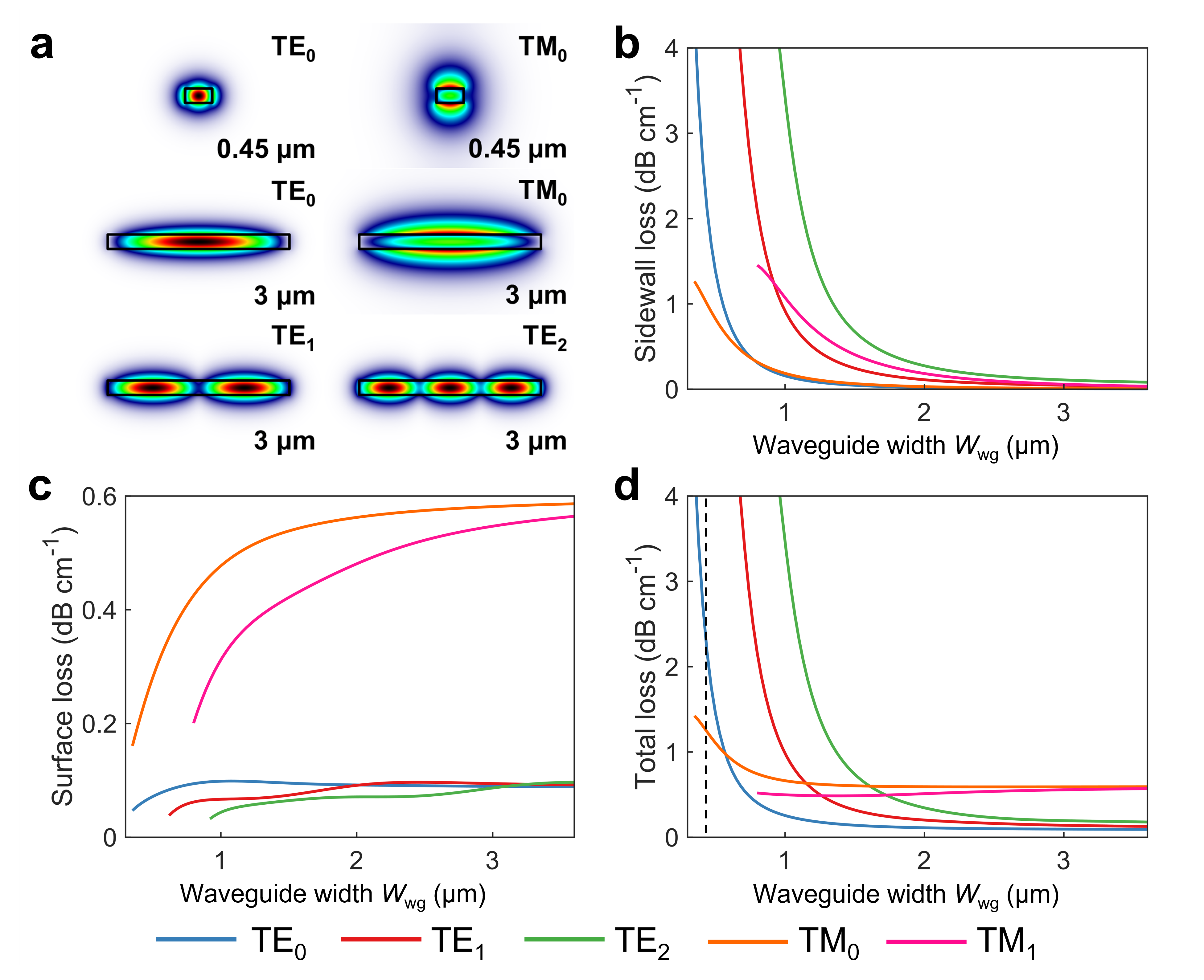


**Figure S10 | Calculated scattering loss for difference modes. a** Calculated field profiles of different modes guided in the singlemode and multimode waveguides. Calculated scattering losses from the sidewalls **b** and surfaces **c**. **d** Total losses for the TE_0_, TE_1_, TE_2_, TM_0_ and TM_1_ modes at varying waveguide width *W*_wg_.

**S7. Additional results for data transmission in the MWS and the MDU**

The data transmissions for the MWS and the MDU with a 5-cm-long waveguide spiral are measured, as shown in Figure. S11. It is observed that the eye diagrams remain open for both individual modes within the MWS and the MDU (with a high ER of > 11.9 dB), indicating negligible crosstalk among different modes within the delay line.


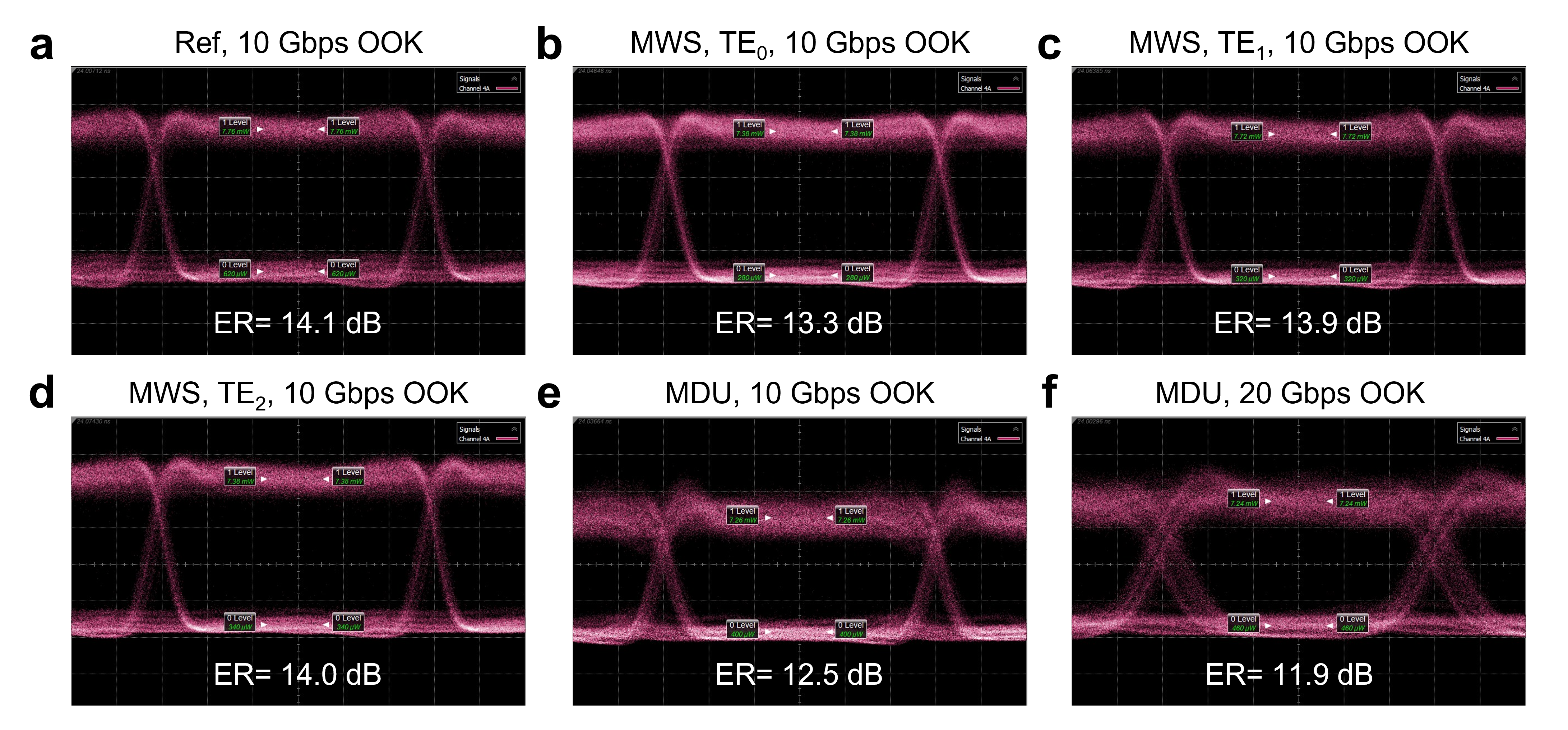


**Figure S11 |** **The** **data transmissions measurement for the MWS and the MDU** **with a 5-cm-long waveguide spiral.** Measured optical eye diagram of 10 Gbps OOK signal for **a** reference straight waveguide, **b-d** TE_0_, TE_1_, TE_2_ in MWS. Measured optical eye diagram of **e** 10 Gbps and **f** 20 Gbps OOK signal for MDU.

# S8. Experimental setup for the delay measurement

Figure S12 illustrates the experimental setup for delay measure by measuring the phase responses and delay of the microwave signals. The input light with an optical power of 13 dBm from a tunable CW laser was polarization controlled and modulated by using a commercial modulator, which was driven by the RF signal from a vector network analyzer (VNA). The modulated optical signal is then coupled to input port of the chip by grating coupler. The time delays of the channels to be tested are controlled by configuring the optical switches on the chip to the desired states with a multi-channel programmable control circuit (PCC). Finally, the output optical signals were converted to RF signals by the PDs and received by the VNA. The phase responses of tunable delay line are read-out and derived to the RF frequency to obtain the delay.


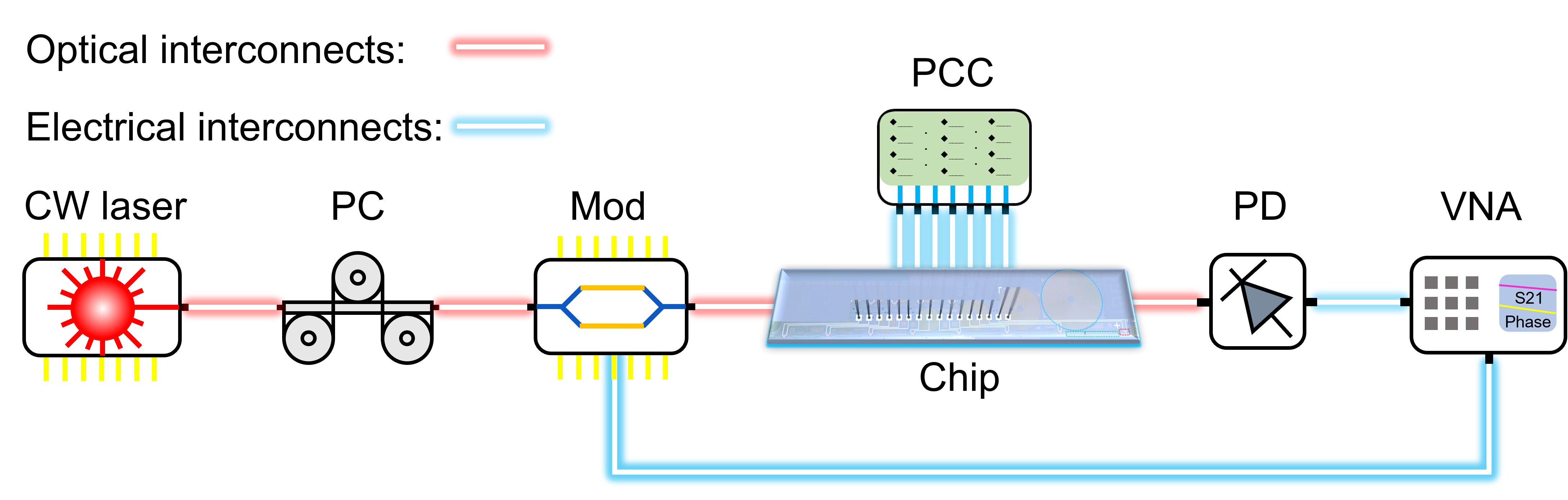


**Figure S12 |** **Schematic configurations of the setup for the delay measurement.** CW, Continuous-wave. PC, polarization controller. Mod, modulator. PD, photodetector. VNA, vector network analyzer. PCC, programming control circuit. The red lines represent optical interconnects, while the blue lines represent electrical interconnects.

**S9. The overall transmission for tunable multimode photonic delay line**

The overall transmissions for the tunable multimode photonic delay line at different delay states are shown in Figure S13. As schematic depicted in Fig. S13(a), distinct delay states can be achieved by selectively routing different optical paths via the control of optical switches. The overall transmission of each individual delay state, as well as the entire tunable multimode delay line have been measured as shown in Fig. S13(b). Here, the measured excess loss for different delay states of the tunable multimode photonic delay line is normalized to the delay reference arm by excluding the losses from the mode DEMUXes/MUXes and optical switches. It is shown that the loss of the tunable multimode delay line increases linearly with the delay time. Moreover, the measured transmissions of the tunable multimode delay line exhibit low ripples across a broad wavelength range for different delay states. The ripples are primarily attributed to the accumulation of low inter-mode crosstalk induced by the structural defects. It is worth noting that the delay loss of the multimode photonic delay line itself is smaller than the loss of 0.004 dB/ps measured for the MDU which includes the mode MUXes/DEMUXes. In order to further enhance the signal quality of multiple modes, subsequent optimizations should be made by e.g., increasing the spiral bending radius, widening the waveguide core, and optimizing the mode MUXes/DEMUXes.


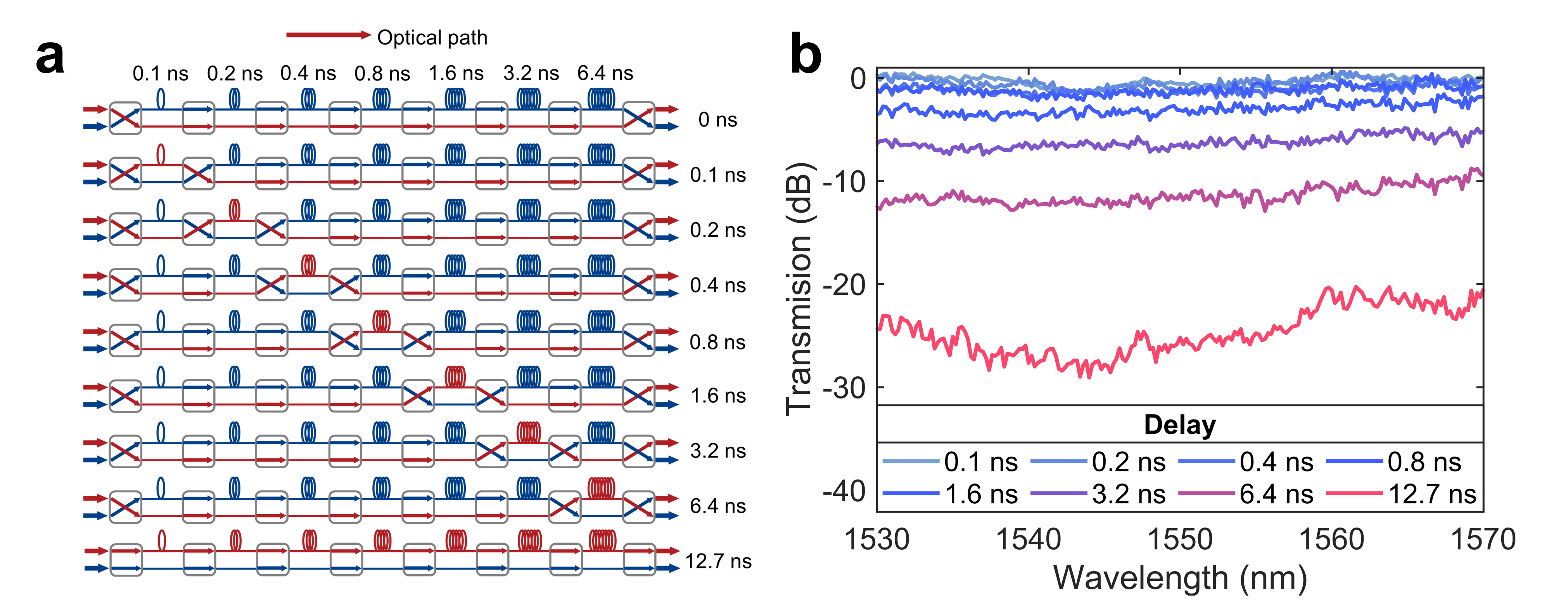


**Figure S13 |** **The measured overall transmissions for** **tunable multimode photonic delay line at different delay states. a** Schematic of the optical path for different delay states. **b** Measured overall transmissions for multimode photonic delay line at different delay states.

**S10. Performance comparison of on-chip tunable** **photonic delay lines**

Table S2. Performance comparison of on-chip tunable photonic delay lines

| Ref | Platform | Bit | Delay range (ps) | Res  (ps) | Line delay density (ps cm^-1^) | WG loss  (dB cm^-1^) | Delay loss (dB ps^-1^) | Footprint (mm^2^) | Delay density  (for single WSs)  (ps mm^-2^) | Delay density  (for TDLs)  (ps mm^-2^) |
| --- | --- | --- | --- | --- | --- | --- | --- | --- | --- | --- |
| Ref ^7^ | SiN | 4 | 12350 | 850 | 51.30 | 0.01 | 0.0002 | 3825 | < 10 | 3.229 |
| Ref ^4^ | SOI | 7 | 1270.0 | 10.0 | 123.7 | 0.90 | 0.0730 | 11.84 | 376.7 | 107.3 |
| Ref ^3^ | SOI | 7 | 1270.0 | 10.0 | 66.67 | 0.60 | 0.0090 | 28.62 | 827.6 | 44.37 |
| Ref ^8^ | SiO_2_ | 4 | 90.200 | 6.00 | 48.36 | 1.26 | 0.0260 | 602.0 | < 10 | 0.1498 |
| Ref ^19^ | SOI | 7 | 191.37 | 1.42 | 125.3 | 2.47 | 0.0197 | 13.32 | 4373 | 14.37 |
| Ref ^2^ | SOI | 10 | 5110.0 | 10.0 | 124.3 | 0.28 | 0.0023 | 12.98 | 3075 | 393.7 |
| Ref ^5^ | TFLN | 4 | 150.00 | 10.0 | 74.80 | 1.72 | 0.0230 | 50.00 | 2126 | 3.000 |
| Ref ^6^ | SiN | 5 | 395.50 | 12.0 | 59.00 | 0.13 | 0.0022 | 35.00 | 1157 | 11.30 |
| **This work** | **SOI** | **7** | **12700** | **100** | **376.9** | **TE_0_: 0.20**  **TE_1_: 0.31**  **TE_2_: 0.49** | **0.0040** | **3.850** | **8200** | **3299** |

Res, resolution. WG, waveguide. WS, waveguide spiral. TDL, tunable delay line. SOI, silicon on insulator. SiN, silicon nitride. SiO2, silica. TFLN, thin film lithium niobate.

**References**

1 Linjie Zhou LZ, Xinyi Wang XW, Liangjun Lu LL, Jianping Chen JC. Integrated optical delay lines: a review and perspective [Invited]. *Chin Opt Lett* 2018; **16**: 101301.

2 Hong S, Zhang L, Wang Y, Zhang M, Xie Y, Dai D. Ultralow-loss compact silicon photonic waveguide spirals and delay lines. *Photon Res* 2022; **10**: 1.

3 Wang X, Zhou L, Li R, Xie J, Lu L, Wu K *et al.* Continuously tunable ultra-thin silicon waveguide optical delay line. *Optica* 2017; **4**: 507.

4 Xie J, Zhou L, Li Z, Wang J, Chen J. Seven-bit reconfigurable optical true time delay line based on silicon integration. *Opt Express* 2014; **22**: 22707.

5 Ke W, Lin Y, He M, Xu M, Zhang J, Lin Z *et al.* Digitally tunable optical delay line based on thin-film lithium niobate featuring high switching speed and low optical loss. *Photon Res* 2022; **10**: 2575.

6 Lin D, Shi S, Cheng W, Liu P, Lu M, Lin T *et al.* A High Performance Silicon Nitride Optical Delay Line With Good Expansibility. *J Lightwave Technol* 2023; **41**: 209–217.

7 Moreira RL, Garcia J, Li W, Bauters J, Barton JS, Heck MJR *et al.* Integrated Ultra-Low-Loss 4-Bit Tunable Delay for Broadband Phased Array Antenna Applications. *IEEE Photon Technol Lett* 2013; **25**: 1165–1168.

8 Song QQ, Hu ZF, Chen KX. Scalable and reconfigurable true time delay line based on an ultra-low-loss silica waveguide. *Appl Opt* 2018; **57**: 4434.

9 Xu H, Liu C, Dai D, Shi Y. Direct-access mode-division multiplexing switch for scalable on-chip multi-mode networks. *Nanophotonics* 2021; **10**: 4551–4566.

10 Zhao W, Peng Y, Cao X, Zhao S, Liu R, Wei Y *et al.* 96-Channel on-chip reconfigurable optical add-drop multiplexer for multidimensional multiplexing systems. *Nanophotonics* 2022; **11**: 4299–4313.

11 Dai D, Li C, Wang S, Wu H, Shi Y, Wu Z *et al.* 10‐Channel Mode (de)multiplexer with Dual Polarizations. *Laser &amp; Photonics Reviews* 2018; **12**: 1700109.

12 Hong S, Zhang L, Wang Y, Zhang M, Xie Y, Dai D. Ultralow-loss compact silicon photonic waveguide spirals and delay lines. *Photon Res* 2022; **10**: 1.

13 Zhao W, Liu R, Zhu M, Guo Z, He J, Li H *et al.* High‐Performance Mode‐Multiplexing Device with Anisotropic Lithium‐Niobate‐on‐Insulator Waveguides. *Laser & Photonics Reviews* 2023; **17**: 2200774.

14 Li C, Jiang X, Hsu Y, Chen G, Chow C, Dai D. Ten-channel mode-division-multiplexed silicon photonic integrated circuit with sharp bends. *Frontiers Inf Technol Electronic Eng* 2019; **20**: 498–506.

15 Dai D, Li C, Wang S, Wu H, Shi Y, Wu Z *et al.* 10‐Channel Mode (de)multiplexer with Dual Polarizations. *Laser &amp; Photonics Reviews* 2018; **12**: 1700109.

16 Bauters JF, Heck MJR, John D, Dai D, Tien M-C, Barton JS *et al.* Ultra-low-loss high-aspect-ratio Si_3N_4 waveguides. *Opt Express* 2011; **19**: 3163.

17 Jared FB. Ultra-Low Loss Waveguides with Application to Photonic Integrated Circuits. 2013.

18 Bauters JF, Heck MJR, John D, Dai D, Tien M-C, Barton JS *et al.* Ultra-low-loss high-aspect-ratio Si_3N_4 waveguides. *Opt Express* 2011; **19**: 3163.

19 Zheng P, Wang C, Xu X, Li J, Lin D, Hu G *et al.* A Seven Bit Silicon Optical True Time Delay Line for Ka-Band Phased Array Antenna. *IEEE Photonics J* 2019; **11**: 1–9.
